# Ephrin-Bs Drive Junctional Downregulation and Actin Stress Fiber Disassembly to Enable Wound Re-epithelialization

**DOI:** 10.1016/j.celrep.2015.09.085

**Published:** 2015-11-05

**Authors:** Robert Nunan, Jessica Campbell, Ryoichi Mori, Mara E. Pitulescu, Wen G. Jiang, Keith G. Harding, Ralf H. Adams, Catherine D. Nobes, Paul Martin

**Affiliations:** 1 Schools of Biochemistry and Physiology & Pharmacology, University of Bristol, Bristol BS8 1TD, UK; 2 Department of Pathology, Nagasaki University, Nagasaki 852-8523, Japan; 3 Max Planck Institute for Molecular Biomedicine, 48149 Muenster, Germany; 4 Faculty of Medicine, University of Muenster, 48149 Muenster, Germany; 5 School of Medicine, Cardiff University, Cardiff CF14 4XN, UK

## Abstract

For a skin wound to successfully heal, the cut epidermal-edge cells have to migrate forward at the interface between scab and healthy granulation tissue. Much is known about how lead-edge cells migrate, but very little is known about the mechanisms that enable active participation by cells further back. Here we show that ephrin-B1 and its receptor EphB2 are both upregulated in vivo, just for the duration of repair, in the first 70 or so rows of epidermal cells, and this signal leads to downregulation of the molecular components of adherens and tight (but not desmosomal) junctions, leading to loosening between neighbors and enabling shuffle room among epidermal cells. Additionally, this signaling leads to the shutdown of actomyosin stress fibers in these same epidermal cells, which may act to release tension within the wound monolayer. If this signaling axis is perturbed, then disrupted healing is a consequence in mouse and man.

## Highlights

• Ephrin-B/EphBs are upregulated in the migrating wound epidermis in mouse and man
• Ephrin-B/EphB signaling drives junction loosening, thus enabling re-epithelialization
• Ephrin-B/EphB signaling also leads to dissolution of stress fibers and tension release
• In human chronic wounds ephrin-Bs are misregulated and may be a therapeutic target

## Graphical Abstract

**Figure:**
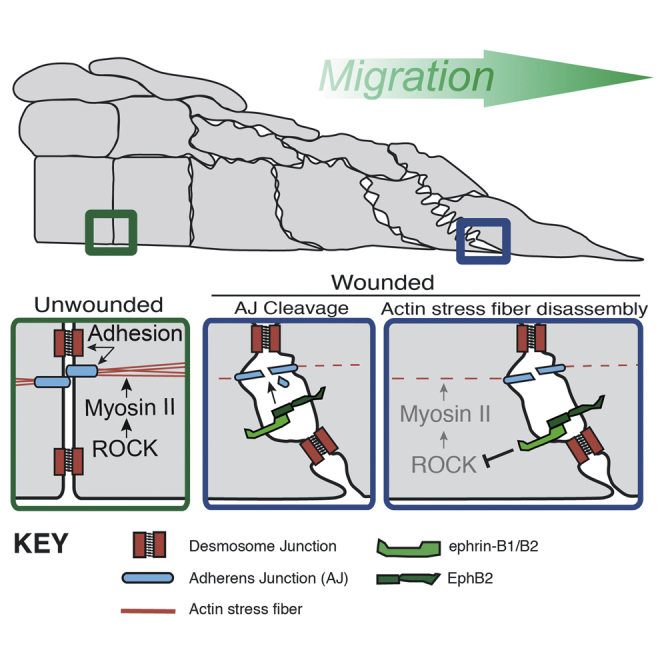

## Teaser

Epithelial migration is essential for skin wound healing. Nunan et al. show that ephrin-B1 is upregulated for the duration of repair and drives both loosening of adherens and tight junctions and release of actomyosin tension to enable re-epithelialization. Misregulation of Eph/ephrin-B signaling in mouse and man considerably hinders healing.

## Introduction

Tissue wounding triggers a robust inflammatory response and results in the rapid but transient plugging of the wound with a fibrin scab, but subsequently the skin must be more permanently sealed to restore its protective function. One key component of this process is re-epithelialization, which involves migration and proliferation of epidermal keratinocytes to cover the denuded surface. Wound-edge epidermal cells upregulate numerous genes as well as reorganize their actin and microtubule cytoskeletons in order to commence migration as a tongue between the wound scab and healthy underlying granulation tissue (Eming et al., 2014). This advancing tongue comprises about 70 rows of cells in a murine skin wound, all of which must dramatically alter their polarity, migratory, and adhesion status. One family of potential regulators of keratinocyte wound migration are the Eph receptors and their ephrin ligands, whose signaling is known to be pivotal in numerous developmental and pathological cell and tissue movements (Adams et al., 1999, Astin et al., 2010, Pasquale, 2008, Poliakov et al., 2004).

Ephs are a large family of tyrosine kinase receptors and are subclassed into EphAs and EphBs, depending on their extracellular sequence homology and binding preference for their ligands, the membrane-bound ephrins (Pasquale, 2004). Generally, the GPI-anchored ephrin-As bind EphA family receptors and the transmembrane ephrin-Bs bind EphBs, but there is some degree of redundancy in this signaling relationship (Gale et al., 1996, Pasquale, 2004). During embryogenesis EphB/ephrin-B signaling has been shown to maintain mesoderm/epidermal interfaces in the developing *Xenopus* embryo (Rohani et al., 2011) and promote retinal progenitor cell migration into the eye field (Moore et al., 2004). In addition, zebrafish studies have shown Eph signaling to be crucial for somite development and specification of boundaries between somites (Barrios et al., 2003, Durbin et al., 1998). EphB/ephrin-B interactions also have been shown to influence development of the vasculature through demarcation of arteries and veins (Adams et al., 1999) and regulation of endothelial cell sprouting (Wang et al., 2010) and motility (Bochenek et al., 2010). Ectopic expression of ephrin-B1 causes cell:cell dissociation of *Xenopus* blastomeres (Jones et al., 1998), and, in the intestinal crypts of the mammalian gut, EphB/ephrin-B signaling maintains correct Paneth cell compartmentalization by regulating differential cell:cell adhesion (Solanas et al., 2011).

Here we show that, immediately following wounding, basal keratinocytes upregulate ephrin-Bs and EphBs, which leads to the dissolution of several classes of adhesion junctions between neighboring epidermal cells, and this loosening enables polarized migration. A second function appears to be to enable wound-edge keratinocytes to disassemble contractile stress fibers and, thus, release epithelial tension. If ephrin-B signaling is disrupted in murine wounds, then re-epithelialization fails and the wound remains open. The same mechanisms appear to be true for human wound re-epithelialization, and this clearly may have important implications for chronic skin wound healing in patients where re-epithelialization fails.

## Results

There is considerable evidence to indicate a role for Eph/ephrin signaling in the regulation of both migration status (Bochenek et al., 2010, Davy et al., 2004, Davy and Soriano, 2007, Moore et al., 2004, Santiago and Erickson, 2002), particularly of cancer cells (Astin et al., 2010, Cortina et al., 2007, Genander, 2012, Pasquale, 2010), and of cell-cell junctional relationships with neighbors (Jones et al., 1998, Solanas et al., 2011), from the observation of various developmental episodes as well as in the retention of stem cells within adult tissue niches (Batlle et al., 2002, Conover et al., 2000, Genander, 2012). For these reasons we chose to investigate the changing expression profiles of Ephs and ephrins following skin wounding, since tissue damage triggers very dramatic migratory responses by several cell lineages as part of the healing process.

Our qPCR studies indicate that prior to wounding many ephrins and Eph receptors are expressed within murine skin; all ephrin-Bs and EphBs are expressed to varying degrees, as are all ephrin-As, but only EphA1, A2, A4, and A7 are expressed at above background levels (Figure S1A).

### Ephrin-B1 and Associated EphB Receptors Are Upregulated following Skin Wounding

**Figure 1:**
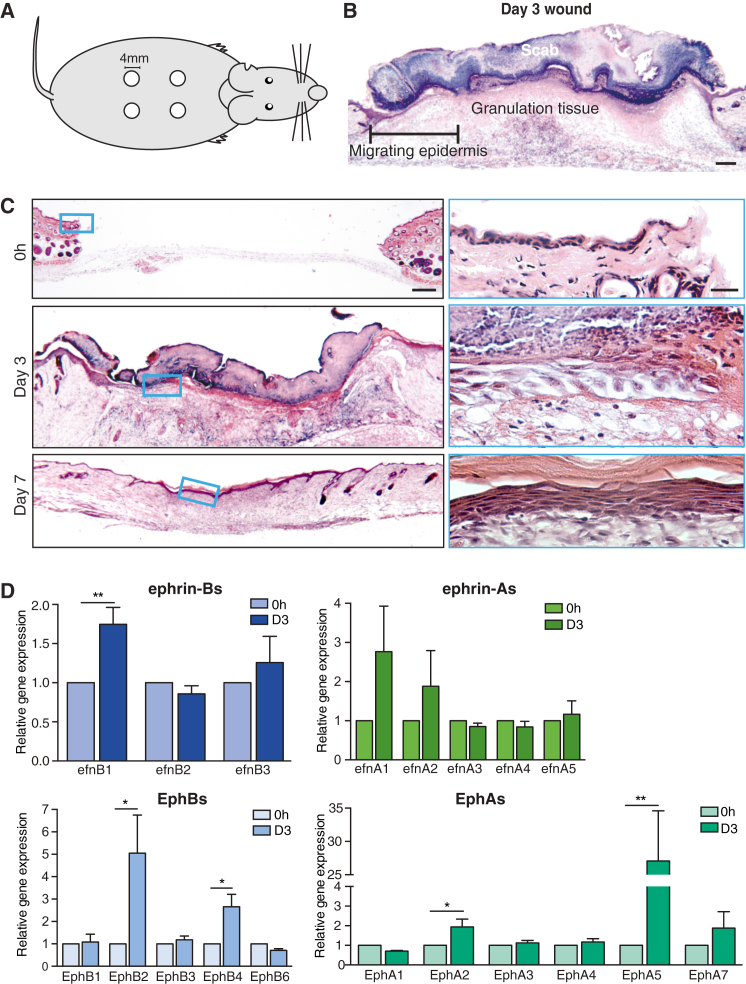
Ephrin-B1 and Associated EphB Receptors Are Upregulated following Skin Wounding (A) Schematic illustrates the location of full-thickness skin wounds (4 × 4mm diameter) made on adult mice. (B) H&E-counterstained image of a day 3 wound section illustrates the extent of epidermal migration. (C) H&E-counterstained sections from wounds at the indicated time points, with magnified insets of the epidermal tongue within the boxed areas, are shown. (D) The qPCR quantification of changes in epidermal ephrin (efnA-green; efnB-blue) and Eph (EphA-green; EphB-blue) gene expression relative to 18S internal control at 3 days post-wounding is shown (^∗^p < 0.05, as determined by an unpaired Student’s t test, n = 4). Scale bars, 200 μm (B and C) and 12.5 μm (C, inset).

We made 4-mm punch biopsy wounds to the shaved backs of 6-week-old male mice (Figures 1A and 1B). These wounds healed with a very reproducible time course so that by 7 days post-wounding they were fully re-epithelialized (Figure 1C). PCR studies indicated significant changes in the expression levels of several Ephs and ephrins in 3-day wounds at a time when re-epithelialization was underway. In particular, we observed ephrin-B1 to be significantly upregulated (and then downregulated post-healing), alongside EphB2 (a recognized receptor for ephrin-B1) and EphB4. EphA2 and EphA5 also were significantly upregulated (Figure 1D; Figure S1B).

**Figure 2:**
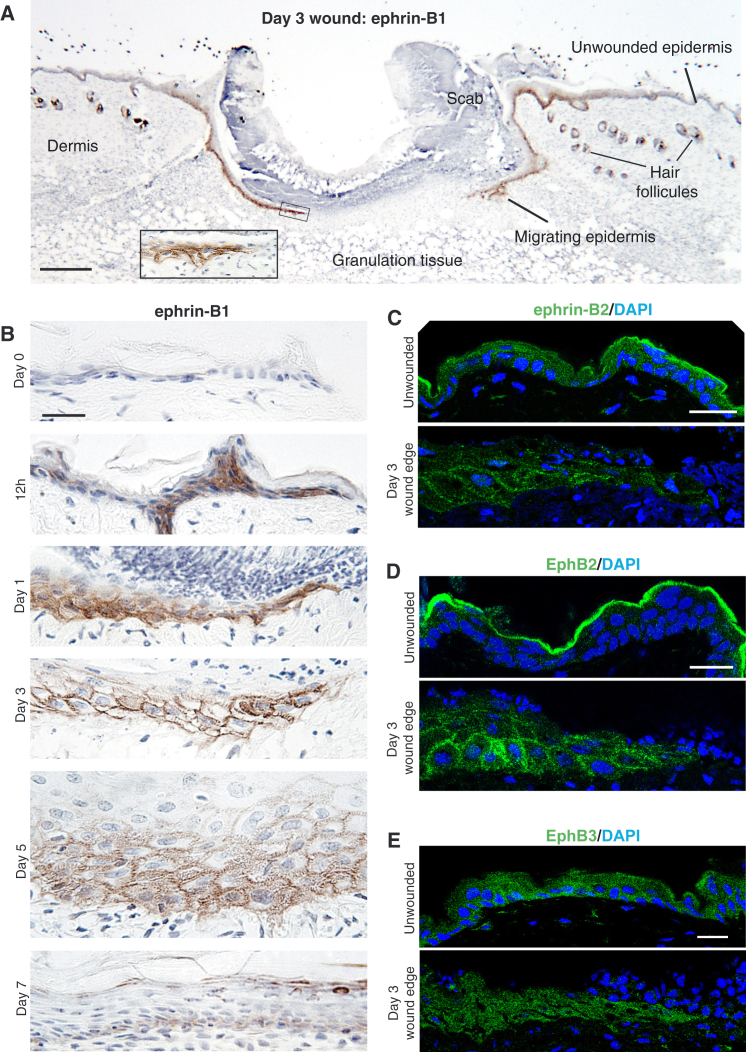
Ephrin-B1 and Associated EphB2 Are Upregulated in the Migrating Epidermis and Re-localized to Cell-Cell Membranes (A) Low magnification of a wild-type day 3 wound showing positive ephrin-B1 immunostaining (brown) in both migrating epidermal tongues and also hair follicles. Inset depicts higher magnification of boxed area. (B) Immunohistochemical time course shows ephrin-B1 expression post-wounding in the epidermis, illustrating upregulation and then translocation to cell margins of basal cells during the period of re-epithelialization and subsequent downregulation when the wound is closed. (C) Immunofluorescence staining shows ephrin-B2, which is homologous to ephrin-B1 and similarly expressed in unwounded and wound-edge epidermis at day 3. (D and E) Immunofluorescence staining shows EphB2 (D) and EphB3 (E), receptors for ephrin-B1/B2 in unwounded versus wound-edge epidermis. Scale bars, 100 μm (A) and 20 μm (B–E).

We further investigated the tissue specificity and spatial localization of upregulated ephrin-B1 by immunohistochemistry (IHC) on wound sections taken from tissues at various time points after wounding (Figures 2A and 2B; Figure S2A). In unwounded skin and until 6 hr post-wounding, we saw ephrin-B1 expression only in hair follicles (Figure 2A; Figure S3D). However, from 12 hr there was clear upregulation, and subsequently from days 1 to 5 we observed translocation of ephrin-B1 protein to the membrane in basal and just suprabasal keratinocytes, extending up to 70 cell rows back from the leading edge. At 7 days, when the wound had fully re-epithelialized but prior to full re-stratification, ephrin-B1 levels and localization reverted to background, unwounded levels (Figure 2B). Because of known binding and signaling redundancy across the ephrin-B/EphBs, we also investigated expression and localization of ephrin-B2 as well as EphB2 and EphB3, which have been shown to function as the chief receptors for ephrin-B1 and -B2 (Gale et al., 1996). Immunostaining for ephrin-B2 showed diffuse expression throughout the epidermal layers, becoming membrane localized after wounding (Figure 2C). The receptors EphB2 and B3 are expressed in the basal layers of the wounded epidermis post-wounding, but only EphB2 shows evidence of membrane localization at 3 days that resolves by 7 days (Figures 2D and 2E; Figure S2B).

### As Wound Epidermal Cells Migrate forward, They Downregulate All but Their Desmosomal Intercellular Links

**Figure 3:**
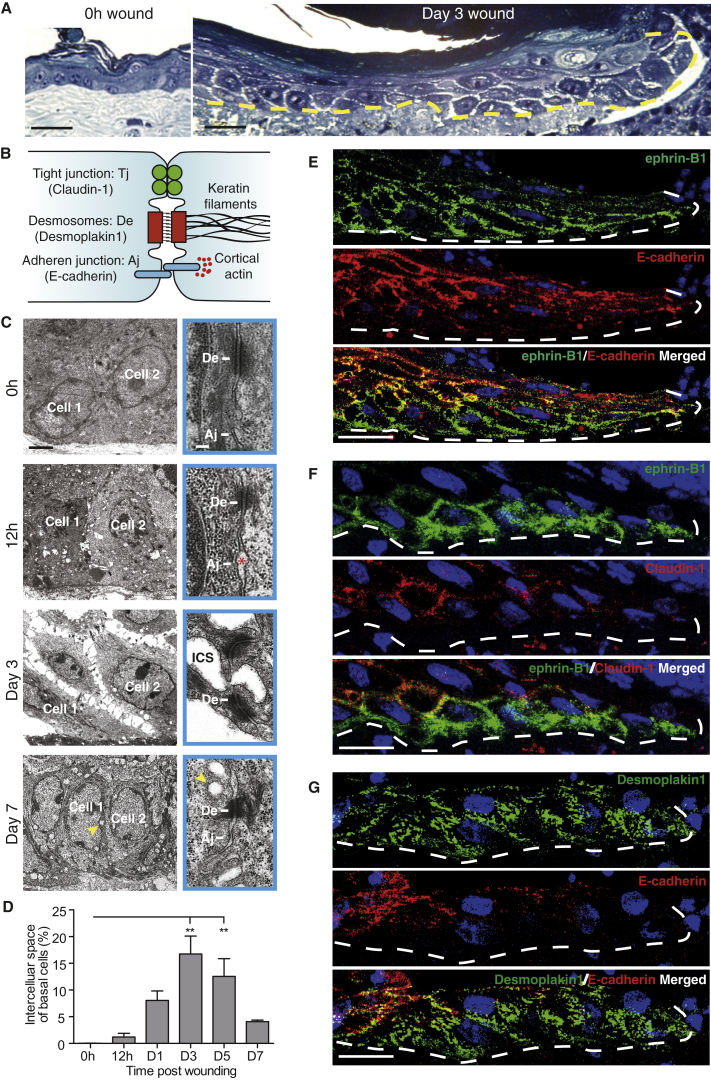
Migrating Epidermal Cells Downregulate All but Their Desmosomal Intercellular Links and Loss of Adhesion Correlates with Increased Ephrin-B1 Expression (A) Resin semi-thin methylene blue-counterstained sections of unwounded and wound-edge epidermis (dermal/epidermal boundary indicated by dotted yellow line) at 0 hr and day 3 post-wounding are shown. (B) Schematic shows three classes of adhering cell:cell junctions found in the epidermis with cytoskeletal linkers and associated molecular markers used in this study. (C) Low- and high-magnification transmission electron microscopy (TEM) time course images of wound-edge basal epidermal cells showing intercellular loosening and junctional status. Early adherens junction loosening at 12 hr is indicated with an asterisk. Clear intercellular loosening is observed at day 3. Intracellular vesicles at day 7 are indicated with a yellow arrowhead. (D) Intercellular spaces at each time point are quantified for a region of epidermis extending 100 μm back from the wound edge (^∗∗^p < 0.01, as determined by one-way ANOVA; n = 3 wounds from three mice). (E–G) Junctional changes in the migrating epidermis at day 3 revealed by immunofluorescence staining. Wherever ephrin-B1 (green, E and F) is upregulated, E-cadherin (red, E) and Claudin-1 (red, F) are downregulated. However, desmosomes marked by desmoplakin1 (green, G) persist in basal ephrin-B1-expressing cells, even when adherens junctions marked by E-cadherin (red, G) are lost. Scale bars, 50 μm (A), 2 μm (C; high-magnification EM, boxed, 0.1 μm), and 20 μm (E–G).

To determine whether ephrin-B/EphB upregulation and junctional relocalization might coincide with any morphological change in wound-edge keratinocytes or their relationship with neighbors, we undertook a histological and electron microscope time course analysis of healing wounds. Comparing unwounded versus wounded sections of skin, we observed an apparent loosening of adhesions between neighboring basal keratinocytes extending back from the leading edge coincident with the zone of ephrin-B1 upregulation (Figure 3A); this loosening appeared to be associated with dissolution of some classes of junctions (see schematic Figure 3B) between neighboring cells. Only finger-like links remained between the advancing keratinocytes of the basal and suprabasal cell layers where we observed ephrin-B1 upregulation.

Our electron microscopy (EM) studies showed that, as well as a spatial correlation, the time course of cell:cell loosening also coincided with ephrin-B1 expression, commencing from 12 hr post-wounding (Figure 3C) and continuing until re-epithelialization was complete. The data show that tight junctions and adherens junctions, which were both present and linking unwounded epidermal cells (Figure 3C; Figures S3A and S3B), were lost between the loosening basal cells, whereas desmosomal junctions were retained and it was these that provided the local bonds that linked finger-like protrusions between neighboring, migrating epidermal cells (Figure 3C). As a means of quantifying cell loosening, we measured intercellular space between basal keratinocytes (Figure 3D). In unwounded epidermis there were no spaces; but, in 12-hr post-wounding sections, small spaces were apparent with a mean area of 1.2% of total basal cell area, and at 3 days the extent of these spaces peaked at 16.7% (Figure 3D). At 7 days, when the epidermal wound fronts had recently met and fused, we saw few spaces between cells but instead numerous sub-membranous vesicles that may have been a consequence of membrane uptake or insertion (Figure 3C).

### Ephrin-B1 Upregulation Largely Correlates with a Reciprocal Downregulation of Both E-cadherin and Claudin-1

Since we observe clear morphological evidence for junctional changes in the migratory epidermal cells, we chose to investigate this further by immunostaining for the molecular components of individual junction sub-types (Figure 3B). Adherens junctions have transmembrane E-cadherin as their central component, linking cells through homophilic binding (Kovacs et al., 2002, van Roy and Berx, 2008). By 3 days post-wounding we see a dramatic diminishment of immunostaining for E-cadherin in basal and immediately suprabasal migrating epidermal cells, again suggesting a reciprocal relationship between ephrin-B expression and junctional maintenance (Figure 3E; Figure S3C). One of the key linker proteins within a tight junction is Claudin-1 and when we immunostain wounds for Claudin-1, we see a reduction of staining which squares with our EM observations suggesting that these junctions are also lost between those epidermal cells that form the advancing migratory sheet (Figure 3F). As expected from our EM studies which show that desmosomes are retained in the migrating epidermis, immunostaining for desmoplakin, which marks desmosomal junctions, is maintained and localized to punctae linking advancing epidermal cells to their neighbors (Figure 3G). After wounds have re-epithelialized at 7 days, E-cadherin and Claudin-1 staining both revert back to that seen in unwounded skin (Figure S3D).

### KO of Ephrin-B1 in the Epidermis Slightly Retards Wound Repair, but KO of Both Ephrin-B1 and Ephrin-B2 Leads to Severe Wound Failure

To test whether this reciprocal correlation between ephrin-B1 upregulation and junctional status within the wound epidermis represents a causal link, we made punch biopsy wounds to the shaved back skin of conditional knockout (KO) mice lacking ephrin-B1 only in the epidermis. Global KO of ephrin-B1 is embryonic lethal (Compagni et al., 2003). In efnB1^Δepi^ mice, we saw a clear absence of ephrin-B1 staining in the hair follicles of unwounded skin (data not shown), as well as in the advancing epidermis (Figure S4A). However, when compared to equivalent wounds in control littermates, we observed only a slight delay in gross wound closure of efnB1^Δepi^ mice. Measuring the extent of epidermal closure at day 2 showed no significant retardation in repair (Figure S4B), and quantification of the gaps between neighboring epidermal cells in EM images of the advancing front (Figure S4C) suggested only minor reduction in the loosening of junctions in KO wounds (Figure S4D), perhaps due to compensation by ephrin-B2 (Figure S4E).

**Figure 4:**
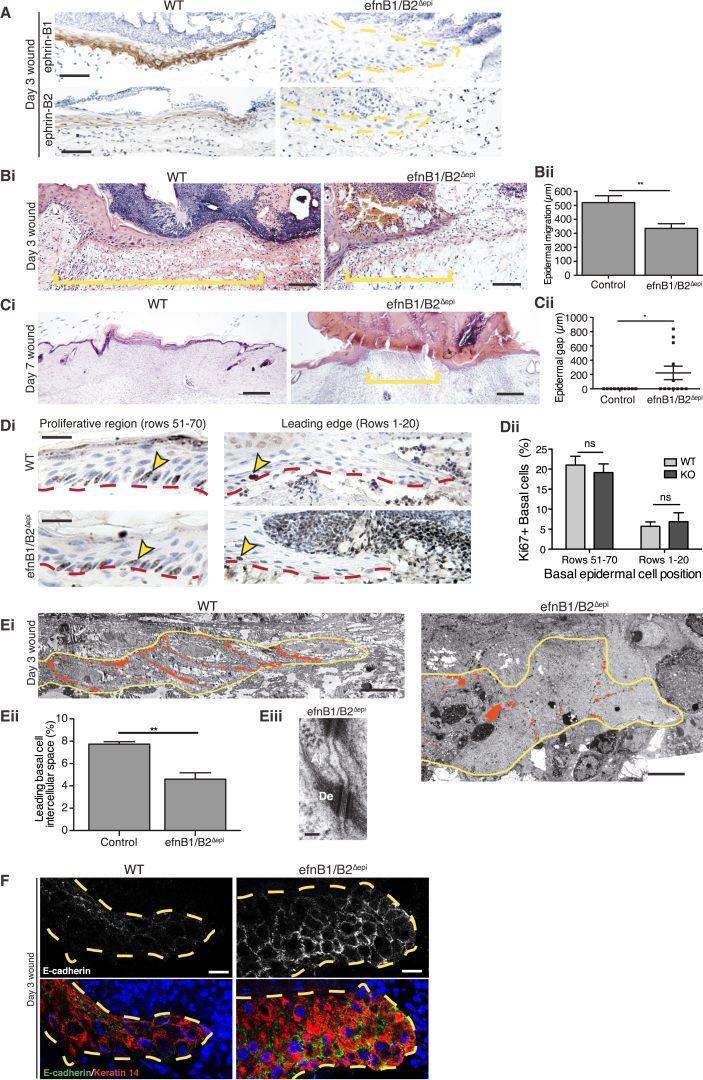
Keratinocyte-Specific KO of Ephrin-B1/B2 Ligands Leads to Severe Wound Failure, Which Correlates with an Increased Presence of Adherens Junctions (A) Immunostaining of day 3 wound edges (indicated by yellow dotted outlines in KOs) confirms the absence of both ephrin-B1 and ephrin-B2 in efnB1/B2^Δepi^ KO mice. (B) (Left) H&E sections of the midregions of day 3 wounds reveal the extent of re-epithelialization in WT versus keratinocyte-specific ephrin-B1/B2 KO mice. Yellow bar indicates length of epidermal tongue used to quantify epithelial migration. (Right) Graphic representation of this data is shown (n = 7 wounds for WT and n = 6 wounds for efnB1/B2^Δepi^ KO; ^∗∗^p < 0.01, as determined by an unpaired Student’s t test). (C) (Left) H&E sections of day 7 wounds of WT versus keratinocyte-specific ephrin-B1/B2 KO mice. Yellow bars indicates distance yet to be re-epithelialized as used to quantify epithelial wound gap. (Right) Graphic representation of this data is shown (n = 12; ^∗^p < 0.05, as determined by an unpaired Student’s t test). (D) (Left) Cell cycle rates in rows 51–70, which overlaps with the epidermal hyperproliferative region, and leading-edge cells where proliferation is generally suppressed (front 20 cells) were quantified by Ki67 immunostaining in day 3 wounds from control versus efnB1/B2^Δepi^ KO mice. Yellow arrowheads indicate typical Ki67-positive nuclei. (Right) Graphic representation of this data is shown (n = 6 wounds for WT and n = 5 wounds for efnB1/B2^Δepi^ KO; neither epidermal zone shows any significant difference between groups as determined by an unpaired Student’s t test). (E) (Top) EM sections of day 3 wounds were used to quantify (bottom left) intercellular spaces (red) in WT versus keratinocyte-specific ephrin-B1/B2 KO epidermal tongues (^∗∗^p < 0.01, as determined by an unpaired Student’s t test; n = 3 wounds from three mice). (Bottom right) High-magnification view shows adherens (Aj) and desmosomal (De) junctions between neighboring efnB1/B2^Δepi^ wound-edge keratinocytes. (F) Immunofluorescence shows the leading epidermal tongue (outlined with yellow dotted line) at day 3 stained for the adherens junction marker E-cadherin (green) and the keratinocyte marker Keratin 14 (red). Scale bars, 50 μm (A), 100 μm (B), 250 μm (C), 20 μm (D), 10 μm (E, top), 100 nm (E, bottom right), and 15 μm (F).

To examine whether the modest difference in wound re-epithelialization between WT and efnB1^Δepi^ mice was a consequence of redundancy across the ephrin-B ligands (Cejalvo et al., 2013, Davy and Soriano, 2007, Orioli et al., 1996), we wounded epidermis-specific, double ephrin-B1 and -B2 KO mice (efnB1/B2^Δepi^), which tissue biopsies indicated to have considerably reduced levels of efnB1 and B2 mRNA (Figures S4F and S4G). These mice have abnormal hair growth (Cejalvo et al., 2013) but otherwise no apparent skin phenotype; however, wound healing appeared severely compromised. Immunostaining confirmed that both ephrin-B1 and ephrin-B2 were indeed absent in the wound epidermis of these mice (Figure 4A). Histology of day 3 wounds showed significant retardation of epidermal migration by comparison to WT healing (mean advancing epidermal tongue length of 336 ± 33.4 μm versus 520 ± 48.8 μm; Figure 4B), with 50% of KO wounds failing to heal by 7 days at a time when 92% of control wounds had fully healed (Figure S4H). Histological analysis of day 7 wounds revealed that all control wounds had fully re-epithelialized while KO wounds remained open (Figure 4C). This defect was not due to changes in cell proliferation, because we observed no significant difference in the percentage of cells cycling between WT and KO in either the hyperproliferative region back from the wound edge or the proliferation-suppressed leading-edge cells (Figure 4D).

### Failure in Wound Re-epithelialization Correlates with a Failure to Dissolve Tight and Adherens Junctions

We investigated junctional status in healing wounds of efnB1/B2^Δepi^ double KO mice by both morphological and molecular means. Our EM studies showed much reduced loosening of junctions between neighboring epidermal cells at 3 days after wounding (Figure 4E), with gaps reduced by 40% of those seen in WT advancing wound epidermis (Figure 4E). Moreover, in these wounds we saw the frequent presence of both adherens junctions (Figure 4E) and tight junctions between neighboring basal epidermal cells, where they were sparse in WT migrating epidermis. Immunostaining of efnB1/B2^Δepi^ KO wounds confirmed our morphological findings with strong positive immunostaining for E-cadherin in basal epidermal cells of the leading tongue, where it was only sporadic in the equivalent WT advancing epidermis (Figure 4F).

These data strongly support our supposition that ephrin-B1 induction is a key wound-activated signal for triggering loosening of basal epidermal junctions to enable forward migration of cells to facilitate repair of the wound gap.

### In Vitro KD of Ephrin-B1/B2 Confirms that Junction Loosening Is Needed for Epithelial Migration

**Figure 5:**
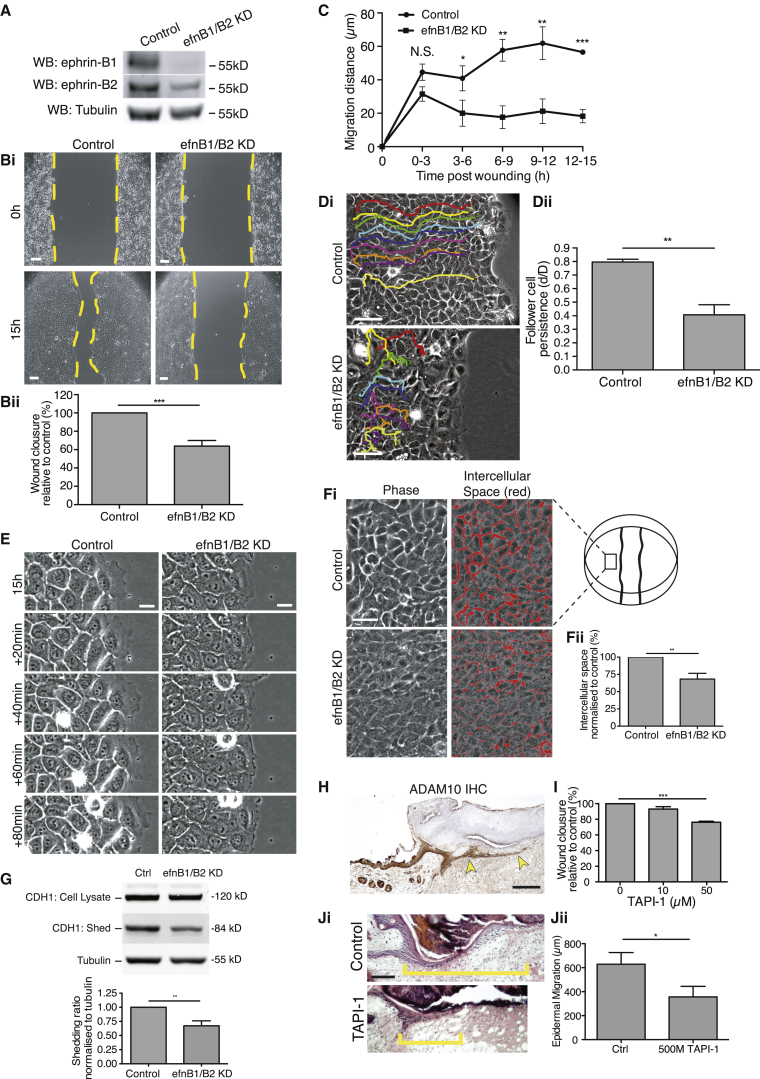
In Vitro KD of Ephrin-B1/B2 Prevents Cell-Cell Loosening and E-cadherin Cleavage (A) Confirmation of ephrin-B1/B2 knockdown (KD) by western blot of human keratinocyte cells (HaCaTs) at 3 days after siRNA transfection is shown. (B) (Top) HaCaT cells transfected with efnB1/B2 siRNA, scratch wounded at confluence, and compared with control wounds at 0 and 15 hr post-scratching (data within each experiment normalized to control). (Bottom) Graph shows relative extent of wound closure at this time point (^∗∗∗^p < 0.001, as determined by an unpaired Student’s t test; n = 6; four wells per experiment). (C) Graph illustrates the rate of HaCaT wound repair from time-lapse imaging over 3-hr periods from 0–15 hr (^∗^p < 0.05, ^∗∗^p < 0.01, and ^∗∗∗^p < 0.001, as determined by an unpaired Student’s t test; n = 4). (D) (Left) Colored tracks of follower cells (fourth row cells) superimposed on phase-contrast images of 15-hr scratch wounds. efnB1/B2 KD leads to considerably reduced persistence of migration. (Right) Graphic representation of follower cell persistence is shown (^∗∗∗^p < 0.01, as determined by an unpaired Student’s t test; n = 4; nine cells per experiment). (E) Snapshot, phase-contrast images show transfected HaCaT cells from 15-hr wounds, taken at 20-min intervals to illustrate how similar are lamellar protrusions of leading-edge cells of KD versus control wounds. (F) (Left) An example of intercellular spaces (red) between cells back from the leading edge (as indicated by wound schematic) at 15 hr post-wounding normalized to control. (Right) Graphic representation of differences in intracellular spaces between control and ephrinB1/B2 KD cells is shown (^∗∗∗^p < 0.001, as determined by an unpaired Student’s t test; n = 3). (G) Western blot shows E-cadherin from culture media and cell lysates (data within each experiment were normalized to tubulin control (^∗∗^p < 0.01, as determined by an unpaired Student’s t test; n = 5). (H) WT day 3 wound shows immunostaining for ADAM10 (brown) in the migrating epidermal tongue (yellow arrowheads). (I) In vitro, 50 μM ADAM10 inhibitor TAPI-1 inhibits HaCaT migration 15 hr after scratch wounding (^∗∗∗^p < 0.001, as determined by one-way ANOVA with Dunnett’s post hoc test; n = 3). (J) (Left) TAPI-1 injected into mouse dorsal skin wounds significantly retards re-epithelialization. Yellow bar indicates length of epidermal tongue. (Right) Graphic representation of the extent of re-epithelialization in control versus TAPI-1-treated wounds is shown (^∗^p < 0.05, as determined by an unpaired Student’s t test; n = 6 wounds from three mice per condition). Scale bars, 100 μm (B), 50 μm (D), 20 μm (E), 50 μm (F), 200 μm (H), and 100 μm (J).

To further analyze the link between ephrin-B signaling and junction dissolution and how this might impact on epidermal sheet migration, we established an in vitro scratch wound assay with HaCaT (human keratinocyte) cells that endogenously express ephrin-B1/B2 localized to cell-cell junctions (Figure S5A). We knocked down ephrin-B1 (91%) and -B2 (74%) using conventional small interfering RNAs (siRNAs) (Figure 5A; Figure S5B) and observed that ephrin-B1/B2 knockdown (KD) scratch wounds had a significantly retarded rate of closure, such that, at 15 hr, repair was 36% less in KD cells compared to control wounds (Figure 5B; Figure S5C). Time course analysis indicated that cell migration stalled from about 3 hr after wounding KD cells, whereas control cells continued advancing forward (Figure 5C; Movie S1). Tracking studies of cells back from the leading edge (follower cells) over 15 hr in the KD wounds showed them to still be moving after 3 hr, but not in a polarized, forward direction (Figure 5D). Stalled wound closure was not a consequence of perturbed lamellipodial assembly in leading-edge cells, because lamellar dynamics appeared identical in control and ephrin-B KD scratch wounds, even after the wound edges had stalled (Figure 5E; Movie S2); neither were there defects in cell polarity (Figure S5D) or proliferation (Figure S5E). Rather, our data suggest that the leading cells might be restrained from moving forward by tension within the epithelial monolayer.

Just as we observed that cell loosening appeared to fail in vivo in ephrin-B KO wounds, the clear bright-phase margins surrounding cells in in vitro epithelial sheets were much reduced after ephrin-B1/B2 KD (Figure 5F), suggestive of tighter adhesions between neighboring cells. To test whether this might be because ephrin-Eph signaling is needed to dissolve adherens junctions, as occured in the gut epithelial cells of intestinal crypts to prevent Paneth cell migration (Solanas et al., 2011), we measured shed E-cadherin in the medium of wounded epithelial sheets and found this to be considerably reduced in the ephrin-B1/B2 KD versus control cells (Figure 5G). These data suggest that ephrin-B1/B2 signaling does indeed lead to cleavage of adherens junctions in the advancing wound epidermis.

Matrix metalloproteinases (MMP) offer one potential mechanism that might link ephrin-B signaling with E-cadherin cleavage (Solanas et al., 2011). Indeed, the MMP ADAM10 was strongly expressed in the migrating epidermis (Figure 5H). We found that inhibition of ADAM10 with TAPI-1 both in vitro (Figure 5I) and in vivo (Figure 5J) inhibited epithelial migration. Furthermore, similar to ephrin-B1 KD, ADAM10 inhibition in vitro resulted in reduced E-cadherin shedding (Figure S5F).

As well as altering cadherin presence at cell junctions, we observed that ephrin-B1/B2 KD also halted the loss of Claudin-1 from junctions between cells of in vitro wounds (Figure S5G).

### Ephrin-B1/B2 Signaling Also May Be Required to Shut Down Actomyosin-Generated Tension within the Advancing Epidermis

**Figure 6:**
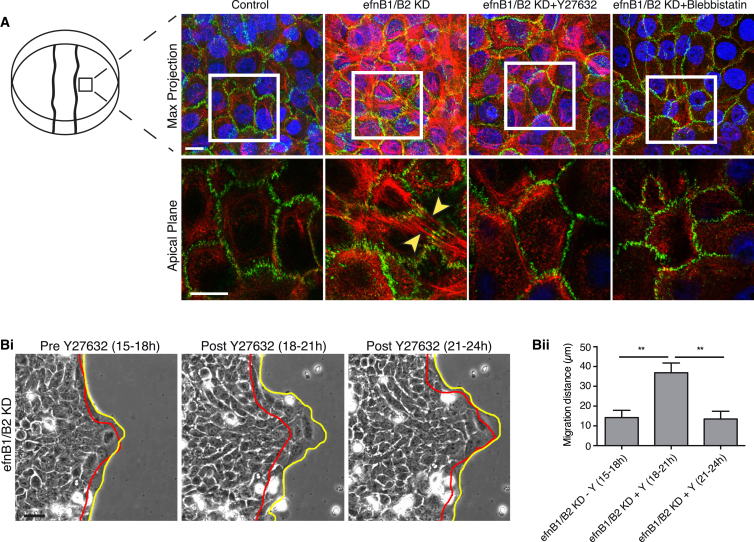
Ephrin-B1/B2 May Be Required to Shut Down Actomyosin-Generated Tension within the Advancing Epidermal Sheet (A) HaCaT cells back from the wound edge after efnB1/B2 KD and subsequent treatment with the ROCK inhibitor Y27632 (650 nM) or the actomyosin inhibitor blebbistatin (10 μM). Cells were stained to reveal actin (red) and E-cadherin (green). A z stack maximum projection is shown with magnification of the apical planes from the boxed area; yellow arrowheads indicate intercellular apical stress fibers linked through adherens junctions. (B) (Left) Time-lapse images of stalled ephrin-B1/B2 KD cells at the start (red) and end (yellow) of 3-hr time windows. Red line indicates position of cell sheet at time of Y27632 addition and yellow line shows wound edge after a 3-hr time period. (Right) Bar chart illustrates migration distance traversed during the 3-hr periods before or after Y27632 addition (^∗∗^p < 0.01, as determined by an unpaired Student’s t test; n = 4). Scale bars, 20 μm (A; 10 μm in apical images, boxed) and 50 μm (B).

From the observations described above, we were curious whether stalling of epithelial migration in ephrin-B1/B2 KD scratch wounds may, in part, be a consequence of unreleased tension within the follower cells further back from the wound edge. At 3 hr post-wounding when stalling was first occurring in ephrin-B1/B2 KD cells, we observed a very different pattern of actin stress fibers in KD versus control follower cells. They exhibited considerably more stress fibers, with many of these extending fully across the cell and linking adherens junctions between cells, whereas most follower cells in control wounds exhibited only cortical actin filaments with very few stress fibers (Figure 6A). This failure to dissolve actin stress fibers in ephrin-B KD wounds was almost completely abrogated by exposure of cells to the ROCK inhibitor Y27632 (Figure 6A), which did not alter junction staining in control cells, and we saw a similar response after treatment with blebbistatin (Figure 6B). Movie analysis of scratch wounds shows that previously stalled ephrin-B KD cells rapidly responded to Y27632 (Figure 6B; Figure S6B; Movie S3) or blebbistatin (Figure S6C) by resurgent forward migration, suggesting that actin-generated tension release (although we have not directly measured it) may be part of the mechanism whereby ephrin-B signaling enables wound re-epithelialization. However, it is noticeable that this rescue was only partial and temporary (Figure 6B) as might be expected, since it only influenced tension loss and did not activate junction dissolution.

### Overexpression of Ephrin-B1 Also Leads to Epithelial Cell Separation and May Be Linked to Some Chronic Wound Pathologies

**Figure 7:**
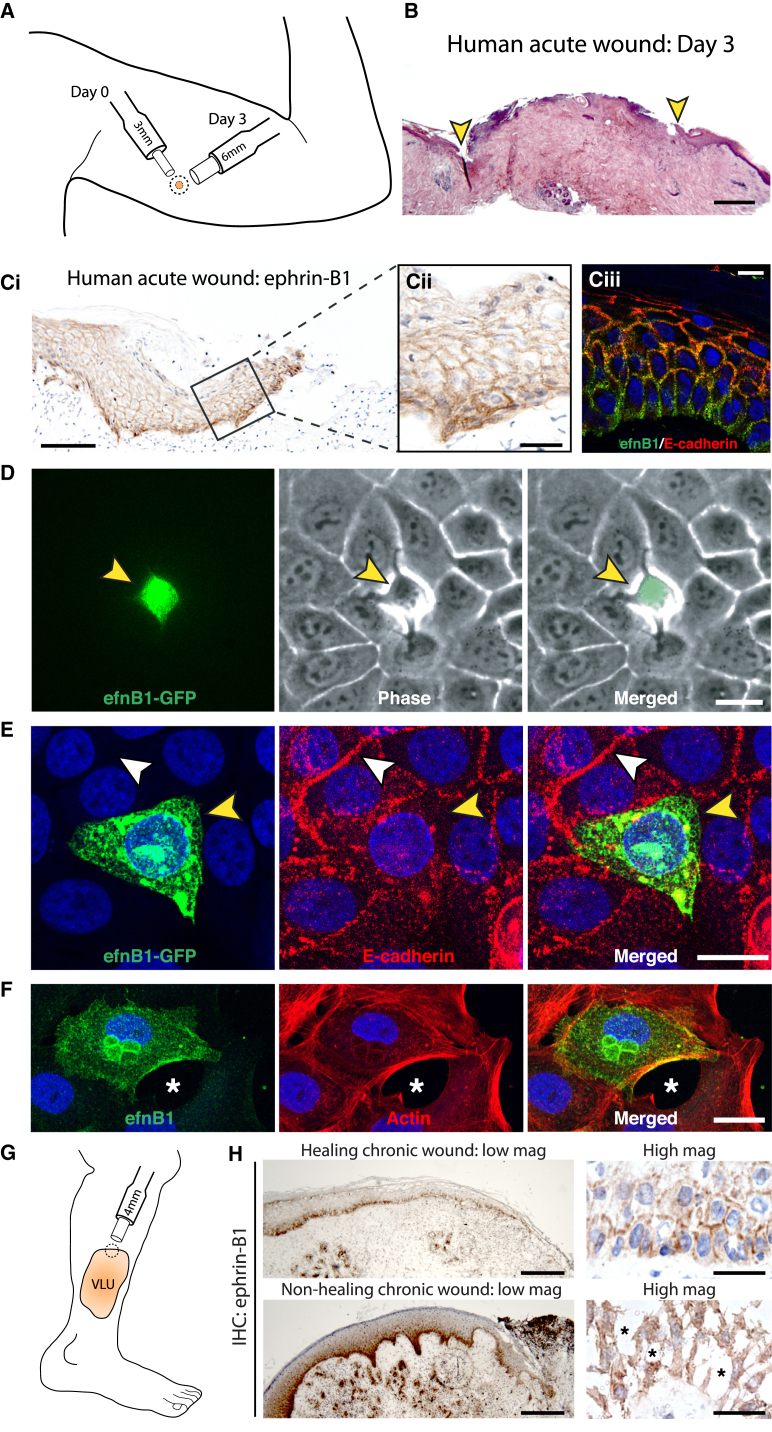
Overexpression of Ephrin-B1 Leads to Cell-Cell Adhesion Breakdown and May Contribute to Impaired Healing in Chronic Wounds (A) Schematic represents location, size, and timing of biopsy wound made in healthy human volunteers. (B) H&E staining of a day 3 human acute wound. Advancing epidermal margins are indicated by yellow arrowheads. (C) Human acute wounds were immunostained for ephrin-B1 as revealed by DAB (brown) and dual immunofluorescent staining of ephrin-B1 and E-cadherin (green and red). (D) Individual cell transfected with ephrin-B1:GFP plasmid (green) within a confluent monolayer of HaCaTs. Yellow arrowhead indicates intercellular loosening. (E) Immunostaining of ephrin-B1:GFP cells for E-cadherin. White and yellow arrowheads indicate present or absent E-cadherin staining, respectively, at the interface between transfected cell and all neighbors. (F) Ephrin-B1-transfected cells just behind the wound edge at 15 hr post-scratching. The fixed wound is stained for ephrin-B1 (green) and actin (red). Asterisk indicates intercellular space. (G) Schematic illustrates the location and size of biopsies taken from the margin of patients’ venous leg ulcers. (H) Typical healing (n = 6 patients) and non-healing (n = 6 patients) chronic-wound biopsies immunostained for ephrin-B1 to reveal intensity of ephrhin-B1 staining (low magnification) and degree of cell detachment (high magnification). Asterisks indicate the large intercellular spaces. Scale bars, 400 μm (B), 100 μm (C, left), 25 μm (C, middle), 10 μm (C, right), 20 μm (D), 20 μm (E), 20 μm (F), 250 μm (H, low magnification), and 25 μm (H, high magnification).

Since ephrin KD had such a dramatic effect on murine wound closure, leading to failure of wound re-epithelialization, we wondered whether defects in Eph-ephrin signaling might be in part causal of defective healing in human patients. Day 3 punch biopsy wounds made on the upper arm skin of normal healthy volunteers (Figures 7A–7C; Figures S7A and S7B) exhibited, as in mouse, epidermal migration that contributed to repair of the wound (Figure 7B). Immunostaining of these wounds revealed a rather similar distribution of ephrin-B1 staining to that of healing murine wounds (Figure 7C), except that expression extended back farther (i.e., 100+ cells back from the wound edge). As in healing mouse wounds, E-cadherin immunostaining appeared largely excluded from the basal layers of epidermis in which ephrin-B1 was expressed (Figure 7C), and resin histology revealed intercellular spaces in the migrating epidermal front (Figure S7B). As a first attempt to investigate a clinical link between Eph-ephrin signaling and wound repair, we undertook a qPCR analysis of ephrin-B1 and associated gene transcripts in human chronic wounds. Venous leg ulcers were stratified according to whether they subsequently healed (n = 20) or not (n = 51) within 3 months after initial referral.

These data show that ephrin-B1 and ephrin-B2 are both significantly upregulated (2.7-fold, p < 0.041 and 2.9-fold, p < 0.036, respectively, Mann-Whitney t test) in those chronic wounds that go on to remain stalled. This may appear somewhat counter to our murine studies, above, where we showed that ephrin-B1/B2 signaling was necessary for healing, but perhaps suggests that ephrinB1/B2 signaling during wound re-epithelialization needs to be very finely balanced: some is needed for driving sufficient loosening of junctions to enable re-epithelialization, but too much might be detrimental to healing.

Since our clinical data indicated that excess ephrin-B1 is associated with a failure to heal, we overexpressed ephrin-B1 in individual epidermal cells (HaCaTs) within a confluent monolayer by lipid-transfecting ephrin-B1:GFP plasmid (Figures 7D and 7E). Time-lapse imaging revealed enhanced phase brightness surrounding ephrin-B1-overexpressing cells, suggesting reduced intercellular adhesion (Figure 7D; Movie S4). Consistent with loss of adhesion to neighbors, ephrin-B1-overexpressing cells exhibited reduced E-cadherin reactivity at sites of cell:cell contact, whereas untransfected cells had E-cadherin-positive junctional boundaries (Figure 7E; Figure S7C). In migrating HaCaT’s, overexpression of ephrin-B1 (Figure 7F; Figure S7D) or ephrin-B2 (Figure S7D) resulted in large intercellular spaces, leading to complete loss of contact with their neighbors, which could explain why excessive ephrin-B1 signaling is detrimental to coherent cell migration. Indeed, more global overexpression of ephrin-B1 led to extrusion of individual cells from the epithelial sheet during the period of scratch wound healing. Immunostaining of chronic wound biopsies from patients with non-healing chronic wounds (Figure 7G) confirmed that these wounds exhibited increased levels of ephrin-B1 (Figure 7H, n = 6). While levels of overexpression cannot be directly compared to our in vitro experiments, this lends further support to the hypothesis that too much ephrin-B signaling might lead to excessive junction dissolution, because we saw evidence of individual detached epithelial cells at the leading edge, which was not seen in equivalent biopsies from healing wounds (Figure 7H).

## Discussion

There has been considerable focus on how leading-edge cells migrate forward to repair a wound, but recent studies in tissue culture scratch wound (Farooqui and Fenteany, 2005, Fenteany et al., 2000, Matsubayashi et al., 2011) and *Drosophila* repair (Razzell et al., 2014) models make it clear that cells back from the front have important, non-passive roles to play too. In this study, we show that ephrin-B1 upregulation in the front 70 or so rows of cells back from the leading edge of an in vivo murine wound is key to mobilizing these cells and enabling efficient wound re-epithelialization. Our data suggest that this signal is necessary for both loosening of adherens junctions and tight junctions, but not desmosomal junctions, and also for releasing tension in the epithelial sheet. There is redundancy in this pathway, because, in order to reveal the requirement for ephrin-B signaling in wound repair, we needed a compound KO of both ephrin-B1 and -B2 ligands; but, this is not unprecedented because the same was true also for revealing a role for ephrin-B1 in thymus development (Cejalvo et al., 2013, Luo et al., 2011) and eyelid closure (Davy and Soriano, 2007).

The cells that express ephrin-B1 after wounding are not confined only to the basal layer but extend one or more layers suprabasally also, as is the case for several other proteins implicated in the epidermal migration process including β1 integrins (Hertle et al., 1992); indeed, there is some evidence in the developing nervous system suggesting that ephrin-B1 might even regulate integrin-mediated adhesions to matrix (Arvanitis et al., 2013). This expression beyond the basal layer may reflect the presumed but still rather poorly understood mechanisms whereby a stratified epithelium moves forward, which labeling studies have shown is not restricted only to basal cells but also involves some degree of rolling forward by suprabasal cells (Danjo and Gipson, 2002, Zhao et al., 2003). Clearly this would entail loosening of adhesions between all participating cell layers, as well as the matrix substratum, and so our observations add weight to this being the mechanism for sheet migration in stratified epithelia.

What might be the molecular signals that trigger ephrin induction in the wound epidermis, and how might Eph-ephrin signaling be linked to junction dissolution and tension release within the epithelial sheet? For induction signals, rather little is known for ephrins. Several Ephs and ephrins have been shown to be induced by hypoxia (Vihanto et al., 2005) and we know that wounds are hypoxic (Chang et al., 1983); it is known that wnt signaling can regulate ephrin-Bs (Batlle et al., 2002), and there is good evidence that wnt signaling pathways are activated in the migrating wound epidermis (Okuse et al., 2005). As for mechanisms by which Eph signaling might mediate junction dissolution, there may be clues from previous studies in the intestinal crypts where loss of E-cadherin-based adhesions led to compartmentalizing and retention of Paneth cells in the stem cell niche; here E-cadherin was shed (as we observed also in wounds) as a consequence of ephrin-B1/EphB interactions, and this clipping of E-cadherin was shown to be mediated by the protease ADAM-10 (Solanas et al., 2011). A similar event might be occurring in the much larger domain of the advancing epidermal tongue of a healing wound, and indeed we show that ADAM10 is expressed by these cells. How this might link to relaxation of epithelial tension by shutting down of stress fibers is less clear, although a recent study showed how the converse is true, that tissue tension generation is dependent on assembly of adherens junctions and independent of desmosomal junctions (Harris et al., 2014).

Ephrin-B signaling is known to stimulate Src signaling (Foo et al., 2006, Palmer et al., 2002), which is upstream of p190-GAP activation that inhibits RhoA-mediated stress fiber formation (Fincham et al., 1999). As Src-mediated phosphorylation of Dock 180 and p130 cas stimulates migration (Cunningham-Edmondson and Hanks, 2009, Feng et al., 2011), ephrin-B upregulation has the potential to simultaneously induce cellular loosening while inhibiting cell tension and driving forward migration. Our studies indicate that desmosomal junctions are retained in the wound epidermis, and indeed they form the key links between neighboring cells, preventing them from migrating as independent cells like a mesenchymal advance. However, these desmosomal links may not be entirely unaltered following wounding; a recent study showed how they too become looser and Ca^2+^ dependent upon wounding, and that this switch is likely to be protein kinase C alpha (PKC-α) dependent because PKC-α−/− mice fail to alter these adhesions and exhibit delayed healing (Thomason et al., 2012).

Clearly the regulation of junctional dissolution must be very finely tuned to enable just sufficient loosening between epithelial cells to allow migration without detaching migrating cells entirely from one another so they cease to be a collectively migrating epithelial sheet. This dissolution of some junctions while retaining other classes of junctions to retain a link to neighboring epithelial cells is reminiscent of the partial epithelial-mesenchymal transition (EMT) previously described to occur during several developmental and pathological episodes, including some cancers (Nieto, 2011). Clearly the balance of adhesion dissolution could go too far and become more of a full EMT, and, indeed, when we compare human chronic wounds that will subsequently heal with those that do not, it appears that ephrin-B1 levels are higher and cells appear more individual/mesenchymal at the wound edge. This may be detrimental to re-epithelialization but also might explain why malignancies, known as Marjolin’s ulcer, can arise at the margins of chronic wounds such as these (Meaume et al., 2013, Onesti et al., 2015).

Our data suggest that Eph/ephrin expression signatures might serve as useful prognostic markers of healthy versus potentially non-healing wounds and that this pathway may serve as a key therapeutic target when considering strategies for re-activating healing in patients with chronic wounds. However, any such modulation of the adhesion status of wound-edge epidermal cells must be cautiously approached to avoid further blocking healing or triggering malignancy.

## Experimental Procedures

### Murine Wound-Healing Experiments

All experiments were conducted with approval from the local ethical review committee at the University of Bristol and in accordance with the UK Home Office regulations. To generate epidermal-specific ephrin-B1/B2 KO mice (efnB1/B2^Δepi^), conditional mutants carrying *loxP-*flanked ephrin-B1 (C57/Bl6-efnB1^Lox/Lox^; Compagni et al., 2003) and *loxP*-flanked ephrin-B2 (C57/Bl6-efnB2^Lox/Lox^; Grunwald et al., 2004) were interbred with C57/Bl6-Tg (K5-Cre; Ramirez et al., 2004) transgenic mice. Littermate K5^Cre/+^, efnB1/B2^Lox/Lox^, or age-matched WT C57/Bl6 mice were used as controls. For wounding, 5- to 7-week-old mice were anesthetized with isoflurane and a 4-mm biopsy punch (Kia Industries) was used on shaved dorsal skin to generate four full-thickness wounds per mouse. Where indicated, 50 μl Pluronic gel with or without the ADAM10 inhibitor TAPI-1 (500 μM) was injected beneath each wound at 0, 24, and 48 hr before harvesting at 72 hr.

### Histology, IHC, and Image Analysis

Mouse wounds harvested at the indicated time points were either formaldehyde fixed (4 hr at 4°C, TAAB) and paraffin embedded (FFPE) or fresh snap frozen in optimal cutting temperature (OCT) compound (Tissue-Tek) on liquid-nitrogen-cooled isopentane. Sections (10 μm) from the center of FFPE wounds were stained with H&E (Gills No. 3; Sigma). Re-epithelialization was quantified with day 3 H&E-stained sections by measuring the distance (in micrometers) the epidermis had migrated over the granulation tissue. The distance between epidermal edges (epidermal gap) was measured from sections of day 7 wounds.

For IHC, FFPE sections were deparaffinized and rehydrated, treated for endogenous peroxidases (3% H_2_O_2_, 10 min), processed for antigen retrieval (Proteinase K, 20 μg/ml, 7 min, Fermentas or heat treat in TE for 1 hr at 70°C), blocked for 30 min with 10% horse serum/0.2% Triton X-100, and incubated with primary antibody (overnight 4°C, see Table S1). Primary antibody was detected as previously described (Bass et al., 2011). Slides were imaged on a Leica Diaplan microscope with a Leica DFC290 camera. For fluorescent IHC (IHC-IF), frozen sections (10 μm) were fixed in formaldehyde (10 min, room temperature [RT]), blocked as above, then incubated with primary antibodies (1 hr RT, see Table S1), fluorescent secondary antibody (1 hr, see Table S1), and mounted in ProLong GOLD (Invitrogen). Images of fluorescent cells were acquired on a confocal SP5.

### Gene Expression Analysis

RNA was extracted from ear notches or skin wound biopsies (whole or dermis-removed) cells using Trizol (Sigma). Briefly, RNA (5 μg) was treated with DNase (Roche); cDNA was synthesized using DNase-treated RNA (2 μg) (Maxima, Fermentas). The qPCR was performed using cDNA (10 ng/reaction), Maxima SYBR green (Fermentas), and QuantiTect primers (QIAGEN, Table S2). Relative gene expression was quantified using the delta-delta Ct method (Bustin, 2000).

### Transmission Electron Microscopy and In Vivo Intercellular Space Analysis

Wounds were biopsied at the indicated time points and fixed in 2.5% Gluteraldeyhyde/0.1 M Cacodylate buffer for 2 hr at RT followed by a secondary Osmium Tetroxide fix (1%, 1 hr), then dehydrated through an ethanol series into propylene oxide (PPO) before embedding in resin. Blocks were trimmed and semi-thin (1-μm) sections were counterstained with methylene blue. Ultrathin (0.02-μm) sections from transmission electron microcopy (TEM) were counterstained with lead and 3% Uranyl Acetate, then imaged on a Tecnai 12-FEI 120 kV BioTwin Spirit transmission electron microcope. Intercellular space surrounding the leading-edge basal epidermal cells at the wound edge was quantified from TEM images. Percentage intercellular space was calculated as a function of the total area of leading-edge cells using ImageJ.

### Collection of Human Wound Tissue Immunohistochemical Analyses

For normal acute wound clinical material, we made polo wounds in the upper arm skin of healthy volunteers. The 3-mm punch biopsy wounds were made to locally anesthetized skin and 3 days later these wounds were harvested with a second 6-mm biopsy, which was immediately flash frozen or fixed in formaldehyde for paraffin embedding. This study was supported by an ethical approval from the Local Regional Ethical committee (CUWHRU_13_01). Fresh-frozen biopsies of chronic venous leg ulcers were obtained from patients attending specialist wound-healing clinics following full written informed consent and supported by ethical approval from the Local Regional Ethical Committee (references 04/WSE02/10 and 09/WSE02/59). Biopsies were obtained from the periphery of chronic wounds using a 6-mm punch biopsy after infiltration with local anesthetic (1% lidocaine). Frozen sections of tissues were cut using a Leica cryostat. Multiple sections were divided into two groups as follows: one group was used for immunohistological analysis; and the remaining sections were combined and homogenized using the Ultra-Turrax T8 (IKA Labortechnik) in RNA extraction buffer (ABgene Advanced Biotechnologies) for RNA collection. Our quantitative transcript assay used the Amplifuor detection system (Intergen) as previously described (Jiang et al., 1995, Parr et al., 2004). Each reaction contained half of the 2× concentrated Hotstart Q-master mix (ABgene), 1 pmol forward and reverse primer (see Table S2 for sequences), 10 pmol of the FAM-tagged universal Z probe (Intergen), and the cDNA. The following conditions were used: an initial 12-min denaturing step (94°C), followed by 60 cycles of denaturing at 94°C for 15 s, annealing at 55°C for 40 s, and extension at 72°C for 20 s. The transcript copy number was calculated using an internal standard that was simultaneously amplified with the samples.

### Human Wound IHC

Frozen sections of human wounds were fixed in 4% formaldehyde (TAAB), treated for endogenous peroxidases (0.3% H_2_O_2_, 10 min), blocked for 30 min with 10% horse serum/0.2% Triton X-100, and incubated with primary antibody (overnight 4°C, see Table S1). Primary antibody was detected as previously described (Bass et al., 2011).

### Cell Culture and siRNA Loading

HaCaT cells were obtained from Cell Line Services and maintained in DMEM/GlutMAX (Sigma) supplemented with 10% (v/v) fetal bovine serum (FBS, HyClone) and 1% (v/v) penicillin/streptomycin (culture media). Cells were grown at 37°C and 5% CO_2_; siRNA oligonucleotides (detailed in Table S3) were transfected into HaCaT cells using RNAiMax (1:500, Invitrogen), according to the manufacturer’s instructions. After 48 hr, KD efficiency was determined by western blot using ephrin-B1 (R&D Systems), ephrin-B2 (Sigma), and tubulin antibodies (Serotec) and visualized with fluorescent secondary antibodies (see Table S1). Membranes were imaged with an Odyssey (LI-COR Biosciences) and levels of protein in the siRNA groups were normalized to control using tubulin as the loading reference.

### Scratch Wound Assay

Transfected cells were plated to confluence on Primaria 24-well dishes (Corning) in culture media. Then, 6 hr post-plating, cells were scratch wounded with a trimmed rubber cell scraper to generate an ∼500-μm-width wound. Where indicated, the ADAM10 inhibitor TAPI-1 (50 μM) was added to untransfected cells. The same field of view was imaged (5× Zeiss Axiovert 200M microscope and Orca-ER camera, Hamamatsu) at 0 and 15 hr post-wounding, and the percentage wound closure was normalized to control using Volocity software (Improvision). For determining cell polarity, control or ephrin-B1/B2 KD cells were fixed in ice-cold methanol 15 hr after wounding and stained for the centrosome marker gamma tubulin. Leading edge or follower cells (four rows back) with centrosomes located in the front 120° (tridrant) of the cell, relative to the wound, were considered polarized. For in vitro proliferation measurements, control or ephrin-B1/B2 KD cells were fixed in 4% formaldehyde 15 hr after wounding and stained for the mitosis-specific marker phospho-histone H3, pH3.

### Time-Lapse Imaging

Scratch-wounded HaCaT cells grown on 3.5-cm Primaria culture dishes (Corning) were imaged on a Zeiss Axiovert 200M microscope with an Orca-ER camera (Hamamatsu) camera using Improvision software at 37°C and a 5% CO_2_ feed. Sheet migration distance and individual cell persistence were calculated via manual tracking from time-lapse stills (MTrackJ/ImageJ). For migration rescue experiments, cells were imaged for 3 hr before and up to 6 hr after the addition of Y27632 or blebbistatin.

### In Vitro Intercellular Space Analysis

HaCaT cells transfected with siRNAs were scratch wounded and imaged after 15 hr. As a measure of intercellular space, the extent of phase-bright margins surrounding cells within the monolayer was determined using threshold analysis in ImageJ. Percentage intercellular space was calculated as a function of the total area using ImageJ.

### E-cadherin Shedding Studies

Transfected cells were plated at 30,000 cells/cm^2^ for 24 hr in 3.5-cm dishes. The culture media were replaced with DMEM/GlutMAX/0.5%FBS/1%Pen/Strep (2 ml). After 48 hr, supernatant containing shed E-cadherin was collected and concentrated with a 10-kDa pass AmiconUltra Centrifugal Filter (Millipore). Shed E-cadherin and total cellular E-cadherin were probed by western blotting using an E-cadherin antibody (Sigma). Total and shed E-cadherin bands were normalized to tubulin before the ratio of shed E-cadherin and total cellular E-cadherin was compared between control and efnB1/B2 KD groups.

### Immunocytochemistry

HaCaT cells were plated onto acid-washed glass coverslips coated with poly-l-lysine and Coating Matrix (Sigma). Cells fixed in 4% formaldehyde (TAAB) for 10 min at RT, blocked with sodium borohydride (0.5 mg/ml, Sigma), and permeabilized were further blocked with a solution of 10% horse serum/0.2% Triton X-100/PBS for 5 min. Cells were then incubated with primary antibodies for 1 hr followed by secondary antibodies for 1 hr before mounting in ProLong Gold (containing DAPI).

### Ephrin-B1 and Ephrin-B2 Overexpression

HaCaT cells were transfected using Lipofectamine 2000 (1:500) with a pCDNA-ephrin-B1:GFP plasmid (1 μg/ml), pRK5-ephrin-B1 plasmid (1 μg/ml), or pRK5-ephrin-B2 plasmid (1 μg/ml). For time-lapse imaging, cells were plated to confluence for 48 hr and imaged at 1 frame/min for 60 min with a Leica AF6000 microscope. For immunocytochemistry, ephrin-B1- or ephrin-B2-transfected cells were either fixed after 48 hr or plated to confluence, scratch wounded, and stained for E-cadherin or filamentous actin using phalloidin (1:50).

### Genotyping

Genomic DNA was extracted from ear notches using the hot shot method. Primers against K5cre (forward, GCC TGC ATT ACC GGT CGA TGC AAC GA; reverse, GTG GCA GAT GGC GCG GCA ACA CCA TT), efnB1 (forward, TTA GGA CAA AGG GCT CCC CTA GC; WT reverse, TGA CAG CAG GGT GTG GAC TCA CAT; lox reverse, GCC ATC TTG ACA GTG TTG TCT GC), and efnB2 (forward, CTT CAG CAA TAT ACA CAG GAT G; reverse, TGC TTG ATT GAA ACG AAG CCC GA) were amplified by PCR at 65°C for 40 cycles and products ran on a 1.2% agarose gel.

### Statistical Analysis

Unpaired Students’ t test and one-way ANOVA with Neuman-Keuls post hoc test were performed in GraphPad Prism 5.0 software and reported such that ^∗^p < 0.05, ^∗∗^p < 0.01, and ^∗∗∗^p < 0.001. Data were expressed as mean ± SEM.

## Author Contributions

R.N., W.G.J., K.G.H., R.H.A, C.D.N., and P.M. designed the experiments. R.N., J.C., R.M., and M.E.P. performed the experiments. R.N., C.D.N., and P.M. wrote the paper.

## Supplemental Information

Document S1. Figures S1–S7 and Tables S1–S3

Movie S1. Time-Lapse Imaging of Control (top) and Ephrin-B1/B2 KD (bottom) HaCaT Cells following Scratch Wounding over 15 hr (5 min/frame), Related to Figure 5

Movie S2. Time-Lapse Imaging of Control (top) and Ephrin-B1/B2 KD (bottom) HaCaT Cell Lamellipodia 15 hr after Wounding (2 min/frame), Related to Figure 5

Movie S3. Time-Lapse Imaging of Ephrin-B1/B2 KD HaCaT Cells before and after the Addition of Y27632, Related to Figure 6

Movie S4. Time-Lapse Imaging of a Confluent HaCaT Cell Monolayer with One efnB1-GFP-Overexpressing Cell (green; 1 min/frame) in View, Related to Figure 7

Document S2. Article plus Supplemental Information

## References

[bib1] Adams R.H., Wilkinson G.A., Weiss C., Diella F., Gale N.W., Deutsch U., Risau W., Klein R. (1999). Roles of ephrinB ligands and EphB receptors in cardiovascular development: demarcation of arterial/venous domains, vascular morphogenesis, and sprouting angiogenesis. Genes Dev..

[bib2] Arvanitis D.N., Béhar A., Tryoen-Tóth P., Bush J.O., Jungas T., Vitale N., Davy A. (2013). Ephrin B1 maintains apical adhesion of neural progenitors. Development.

[bib3] Astin J.W., Batson J., Kadir S., Charlet J., Persad R.A., Gillatt D., Oxley J.D., Nobes C.D. (2010). Competition amongst Eph receptors regulates contact inhibition of locomotion and invasiveness in prostate cancer cells. Nat. Cell Biol..

[bib4] Barrios A., Poole R.J., Durbin L., Brennan C., Holder N., Wilson S.W. (2003). Eph/Ephrin signaling regulates the mesenchymal-to-epithelial transition of the paraxial mesoderm during somite morphogenesis. Curr. Biol..

[bib5] Bass M.D., Williamson R.C., Nunan R.D., Humphries J.D., Byron A., Morgan M.R., Martin P., Humphries M.J. (2011). A syndecan-4 hair trigger initiates wound healing through caveolin- and RhoG-regulated integrin endocytosis. Dev. Cell.

[bib6] Batlle E., Henderson J.T., Beghtel H., van den Born M.M., Sancho E., Huls G., Meeldijk J., Robertson J., van de Wetering M., Pawson T., Clevers H. (2002). Beta-catenin and TCF mediate cell positioning in the intestinal epithelium by controlling the expression of EphB/ephrinB. Cell.

[bib7] Bochenek M.L., Dickinson S., Astin J.W., Adams R.H., Nobes C.D. (2010). Ephrin-B2 regulates endothelial cell morphology and motility independently of Eph-receptor binding. J. Cell Sci..

[bib8] Bustin S.A. (2000). Absolute quantification of mRNA using real-time reverse transcription polymerase chain reaction assays. J. Mol. Endocrinol..

[bib9] Cejalvo T., Munoz J.J., Tobajas E., Fanlo L., Alfaro D., García-Ceca J., Zapata A. (2013). Ephrin-B-dependent thymic epithelial cell-thymocyte interactions are necessary for correct T cell differentiation and thymus histology organization: relevance for thymic cortex development. J. Immunol..

[bib10] Chang N., Goodson W.H., Gottrup F., Hunt T.K. (1983). Direct measurement of wound and tissue oxygen tension in postoperative patients. Ann. Surg..

[bib11] Compagni A., Logan M., Klein R., Adams R.H. (2003). Control of skeletal patterning by ephrinB1-EphB interactions. Dev. Cell.

[bib12] Conover J.C., Doetsch F., Garcia-Verdugo J.M., Gale N.W., Yancopoulos G.D., Alvarez-Buylla A. (2000). Disruption of Eph/ephrin signaling affects migration and proliferation in the adult subventricular zone. Nat. Neurosci..

[bib13] Cortina C., Palomo-Ponce S., Iglesias M., Fernández-Masip J.L., Vivancos A., Whissell G., Humà M., Peiró N., Gallego L., Jonkheer S. (2007). EphB-ephrin-B interactions suppress colorectal cancer progression by compartmentalizing tumor cells. Nat. Genet..

[bib14] Cunningham-Edmondson A.C., Hanks S.K. (2009). p130Cas substrate domain signaling promotes migration, invasion, and survival of estrogen receptor-negative breast cancer cells. Breast Cancer.

[bib15] Danjo Y., Gipson I.K. (2002). Specific transduction of the leading edge cells of migrating epithelia demonstrates that they are replaced during healing. Exp. Eye Res..

[bib16] Davy A., Soriano P. (2007). Ephrin-B2 forward signaling regulates somite patterning and neural crest cell development. Dev. Biol..

[bib17] Davy A., Aubin J., Soriano P. (2004). Ephrin-B1 forward and reverse signaling are required during mouse development. Genes Dev..

[bib18] Durbin L., Brennan C., Shiomi K., Cooke J., Barrios A., Shanmugalingam S., Guthrie B., Lindberg R., Holder N. (1998). Eph signaling is required for segmentation and differentiation of the somites. Genes Dev..

[bib19] Eming S.A., Martin P., Tomic-Canic M. (2014). Wound repair and regeneration: mechanisms, signaling, and translation. Sci. Transl. Med..

[bib20] Farooqui R., Fenteany G. (2005). Multiple rows of cells behind an epithelial wound edge extend cryptic lamellipodia to collectively drive cell-sheet movement. J. Cell Sci..

[bib21] Feng H., Hu B., Liu K.W., Li Y., Lu X., Cheng T., Yiin J.J., Lu S., Keezer S., Fenton T. (2011). Activation of Rac1 by Src-dependent phosphorylation of Dock180(Y1811) mediates PDGFRα-stimulated glioma tumorigenesis in mice and humans. J. Clin. Invest..

[bib22] Fenteany G., Janmey P.A., Stossel T.P. (2000). Signaling pathways and cell mechanics involved in wound closure by epithelial cell sheets. Curr. Biol..

[bib23] Fincham V.J., Chudleigh A., Frame M.C. (1999). Regulation of p190 Rho-GAP by v-Src is linked to cytoskeletal disruption during transformation. J. Cell Sci..

[bib24] Foo S.S., Turner C.J., Adams S., Compagni A., Aubyn D., Kogata N., Lindblom P., Shani M., Zicha D., Adams R.H. (2006). Ephrin-B2 controls cell motility and adhesion during blood-vessel-wall assembly. Cell.

[bib25] Gale N.W., Holland S.J., Valenzuela D.M., Flenniken A., Pan L., Ryan T.E., Henkemeyer M., Strebhardt K., Hirai H., Wilkinson D.G. (1996). Eph receptors and ligands comprise two major specificity subclasses and are reciprocally compartmentalized during embryogenesis. Neuron.

[bib26] Genander M. (2012). Eph and ephrins in epithelial stem cell niches and cancer. Cell Adhes. Migr..

[bib27] Grunwald I.C., Korte M., Adelmann G., Plueck A., Kullander K., Adams R.H., Frotscher M., Bonhoeffer T., Klein R. (2004). Hippocampal plasticity requires postsynaptic ephrinBs. Nat. Neurosci..

[bib28] Harris A.R., Daeden A., Charras G.T. (2014). Formation of adherens junctions leads to the emergence of a tissue-level tension in epithelial monolayers. J. Cell Sci..

[bib29] Hertle M.D., Kubler M.D., Leigh I.M., Watt F.M. (1992). Aberrant integrin expression during epidermal wound healing and in psoriatic epidermis. J. Clin. Invest..

[bib30] Jiang W.G., Hiscox S., Hallett M.B., Scott C., Horrobin D.F., Puntis M.C.A. (1995). Inhibition of hepatocyte growth factor-induced motility and in vitro invasion of human colon cancer cells by gamma-linolenic acid. Br. J. Cancer.

[bib31] Jones T.L., Chong L.D., Kim J., Xu R.H., Kung H.F., Daar I.O. (1998). Loss of cell adhesion in Xenopus laevis embryos mediated by the cytoplasmic domain of XLerk, an erythropoietin-producing hepatocellular ligand. Proc. Natl. Acad. Sci. USA.

[bib32] Kovacs E.M., Ali R.G., McCormack A.J., Yap A.S. (2002). E-cadherin homophilic ligation directly signals through Rac and phosphatidylinositol 3-kinase to regulate adhesive contacts. J. Biol. Chem..

[bib33] Luo H., Charpentier T., Wang X., Qi S., Han B., Wu T., Terra R., Lamarre A., Wu J. (2011). Efnb1 and Efnb2 proteins regulate thymocyte development, peripheral T cell differentiation, and antiviral immune responses and are essential for interleukin-6 (IL-6) signaling. J. Biol. Chem..

[bib34] Matsubayashi Y., Razzell W., Martin P. (2011). ‘White wave’ analysis of epithelial scratch wound healing reveals how cells mobilise back from the leading edge in a myosin-II-dependent fashion. J. Cell Sci..

[bib35] Meaume S., Fromantin I., Teot L. (2013). Neoplastic wounds and degenerescence. J. Tissue Viability.

[bib36] Moore K.B., Mood K., Daar I.O., Moody S.A. (2004). Morphogenetic movements underlying eye field formation require interactions between the FGF and ephrinB1 signaling pathways. Dev. Cell.

[bib37] Nieto M.A. (2011). The ins and outs of the epithelial to mesenchymal transition in health and disease. Annu. Rev. Cell Dev. Biol..

[bib38] Okuse T., Chiba T., Katsuumi I., Imai K. (2005). Differential expression and localization of WNTs in an animal model of skin wound healing. Wound Repair Regen..

[bib39] Onesti M.G., Fino P., Fioramonti P., Amorosi V., Scuderi N. (2015). Ten years of experience in chronic ulcers and malignant transformation. Int. Wound J..

[bib40] Orioli D., Henkemeyer M., Lemke G., Klein R., Pawson T. (1996). Sek4 and Nuk receptors cooperate in guidance of commissural axons and in palate formation. EMBO J..

[bib41] Palmer A., Zimmer M., Erdmann K.S., Eulenburg V., Porthin A., Heumann R., Deutsch U., Klein R. (2002). EphrinB phosphorylation and reverse signaling: regulation by Src kinases and PTP-BL phosphatase. Mol. Cell.

[bib42] Parr C., Watkins G., Mansel R.E., Jiang W.G. (2004). The hepatocyte growth factor regulatory factors in human breast cancer. Clin. Cancer Res..

[bib43] Pasquale E.B. (2004). Eph-ephrin promiscuity is now crystal clear. Nat. Neurosci..

[bib44] Pasquale E.B. (2008). Eph-ephrin bidirectional signaling in physiology and disease. Cell.

[bib45] Pasquale E.B. (2010). Eph receptors and ephrins in cancer: bidirectional signalling and beyond. Nat. Rev. Cancer.

[bib46] Poliakov A., Cotrina M., Wilkinson D.G. (2004). Diverse roles of eph receptors and ephrins in the regulation of cell migration and tissue assembly. Dev. Cell.

[bib47] Ramirez A., Page A., Gandarillas A., Zanet J., Pibre S., Vidal M., Tusell L., Genesca A., Whitaker D.A., Melton D.W., Jorcano J.L. (2004). A keratin K5Cre transgenic line appropriate for tissue-specific or generalized Cre-mediated recombination. Genesis.

[bib48] Razzell W., Wood W., Martin P. (2014). Recapitulation of morphogenetic cell shape changes enables wound re-epithelialisation. Development.

[bib49] Rohani N., Canty L., Luu O., Fagotto F., Winklbauer R. (2011). EphrinB/EphB signaling controls embryonic germ layer separation by contact-induced cell detachment. PLoS Biol..

[bib50] Santiago A., Erickson C.A. (2002). Ephrin-B ligands play a dual role in the control of neural crest cell migration. Development.

[bib51] Solanas G., Cortina C., Sevillano M., Batlle E. (2011). Cleavage of E-cadherin by ADAM10 mediates epithelial cell sorting downstream of EphB signalling. Nat. Cell Biol..

[bib52] Thomason H.A., Cooper N.H., Ansell D.M., Chiu M., Merrit A.J., Hardman M.J., Garrod D.R. (2012). Direct evidence that PKCα positively regulates wound re-epithelialization: correlation with changes in desmosomal adhesiveness. J. Pathol..

[bib53] van Roy F., Berx G. (2008). The cell-cell adhesion molecule E-cadherin. Cell. Mol. Life Sci..

[bib54] Vihanto M.M., Plock J., Erni D., Frey B.M., Frey F.J., Huynh-Do U. (2005). Hypoxia up-regulates expression of Eph receptors and ephrins in mouse skin. FASEB J..

[bib55] Wang Y., Nakayama M., Pitulescu M.E., Schmidt T.S., Bochenek M.L., Sakakibara A., Adams S., Davy A., Deutsch U., Lüthi U. (2010). Ephrin-B2 controls VEGF-induced angiogenesis and lymphangiogenesis. Nature.

[bib56] Zhao M., Song B., Pu J., Forrester J.V., McCaig C.D. (2003). Direct visualization of a stratified epithelium reveals that wounds heal by unified sliding of cell sheets. FASEB J..

