# Supplementary material for: Ephrin-Bs Drive Junctional Downregulation and Actin Stress Fiber Disassembly to Enable Wound Re-epithelialization
Source: Cell Rep. 2015 Nov 5;13(7):1380–95. doi: 10.1016/j.celrep.2015.09.085 (PMC4660216; doi:10.1016/j.celrep.2015.09.085)
Supplement: Document S1. Figures S1–S7 and Tables S1–S3 [file mmc1.pdf]

Cell Reports

Supplemental Information

Ephrin-Bs Drive Junctional Downregulation and Actin Stress Fiber Disassembly to Enable Wound Re-epithelialization

Robert Nunan, Jessica Campbell, Ryoichi Mori, Mara E. Pitulescu, Wen G. Jiang, Keith G. Harding, Ralf H. Adams, Catherine D. Nobes, and Paul Martin

Supplemental Figures

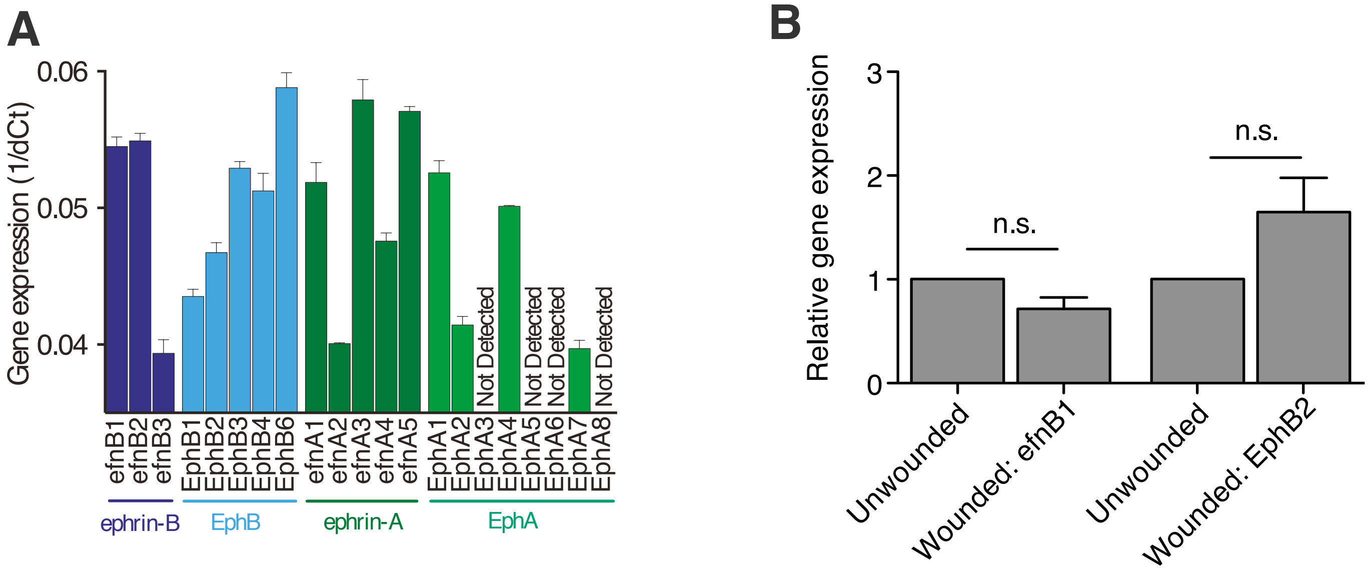

Figure S1, related to Figure 1. Gene expression of Ephs and ephrins in unwounded and wounded adult mouse skin.

(A) Expression levels of Eph receptors and ephrin ligands relative to 18S internal Control in unwounded epidermis (n=3). (B) qPCR quantification of ephrin-B1 and EphB2 expression comparing unwounded and Day 7 wounded (just healed) skin, indicating a return back to baseline levels after repair is complete (n.s P>0.05 as determined by an unpaired Student's t-test, n=2-3).

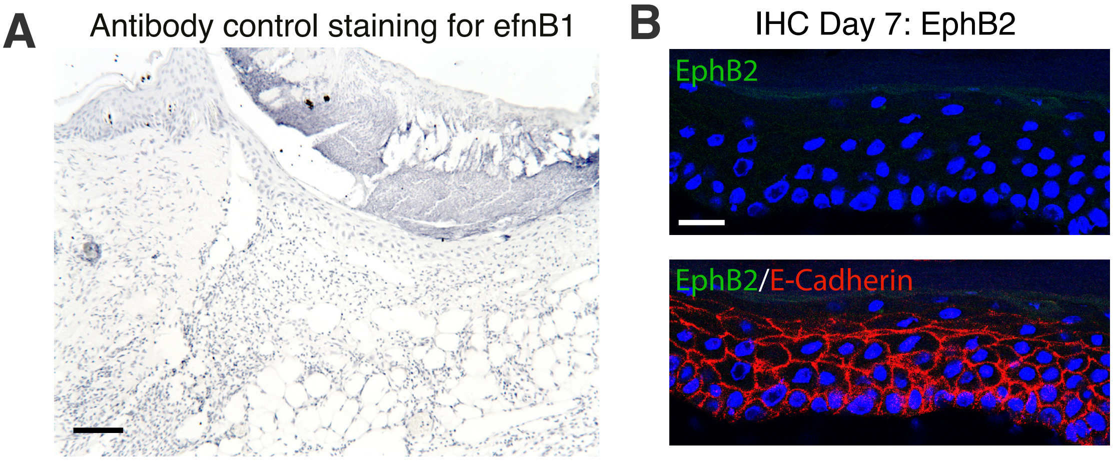

Figure S2, related to Figure 2. Control ephrin-B1 and EphB2/E-cadherin immunostaining in wounds.
(A) No primary antibody (negative) control staining for ephrin-B1 in mouse skin wound. (B) EphB2 and E-cadherin immunostaining in Day 7 wild type wound epidermis to exhibit how these both return to background, pre-wounding expression patterns after the wound has healed. Scale Bars: A = 100µm and B = 25µm.

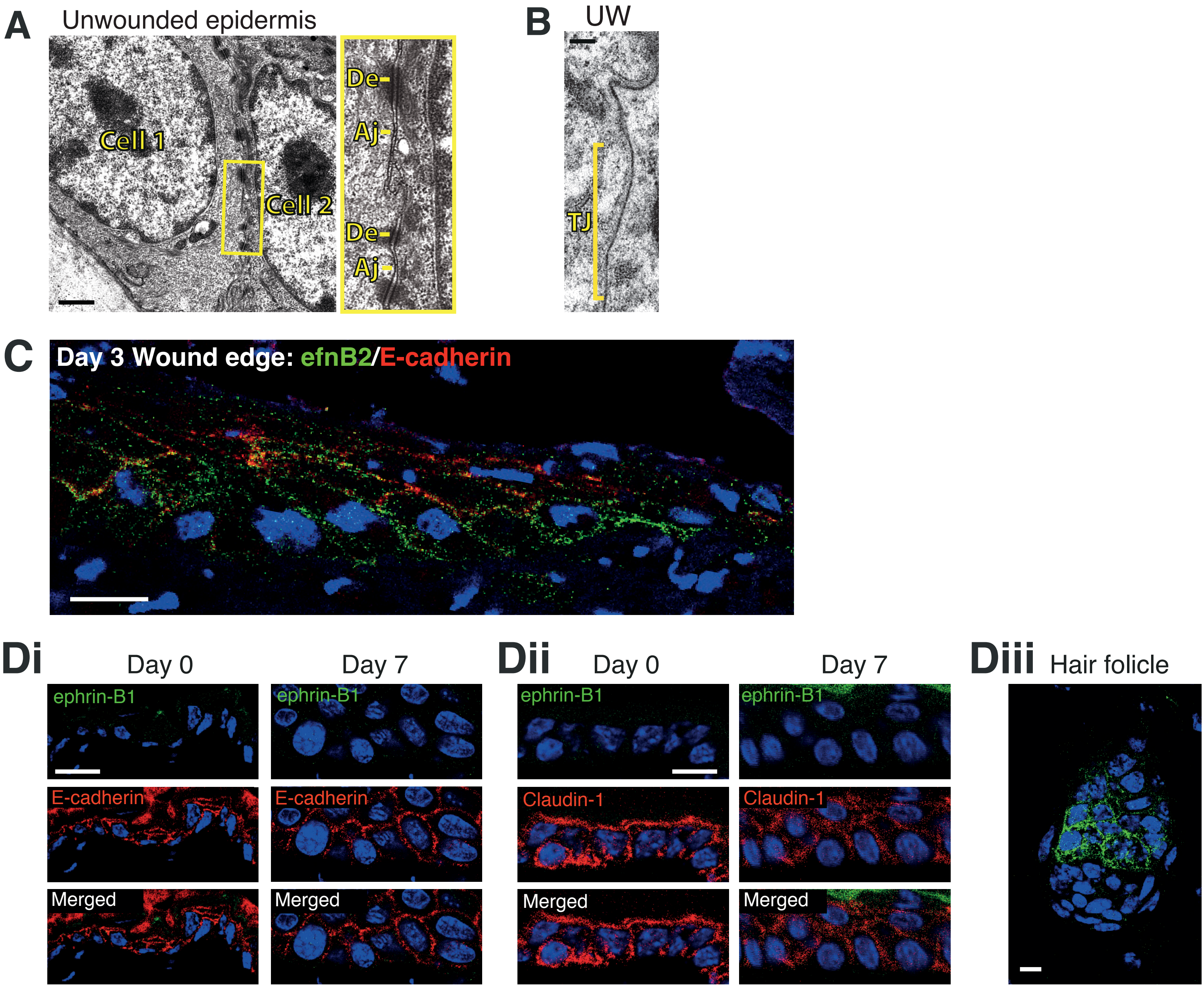

Figure S3, related to Figure 3. ephrin-B1/B2 and junction localization in unwounded and healing epidermis.

(A) TEM images (inset is high magnification view) to indicate desmosomal (De) and adherens (Aj) junctions linking neighbouring epithelial cells in unwounded epidermis. (B) Tight junction (Tj) linking neighbouring cells in unwounded epidermis. (C) Similar to ephrin-B1, ephrin-B2 membrane expression in Day 3 wound epidermis is associated with a down-regulation of E-cadherin immunoreactivity. (D) As ephrin-B1 expression shuts down in Day 7 just healed epidermis, E-cadherin (Di) and Claudin-1 (Dii) expression patterns return to pre-wound distribution. (Diii) Hair follicles constitutively express ephrin-B1 and function as ephrin-B1 positive control staining for Di and Dii. Scale Bars: A = 1µm; B = 100nm; C = 25µm; D = 10µm.

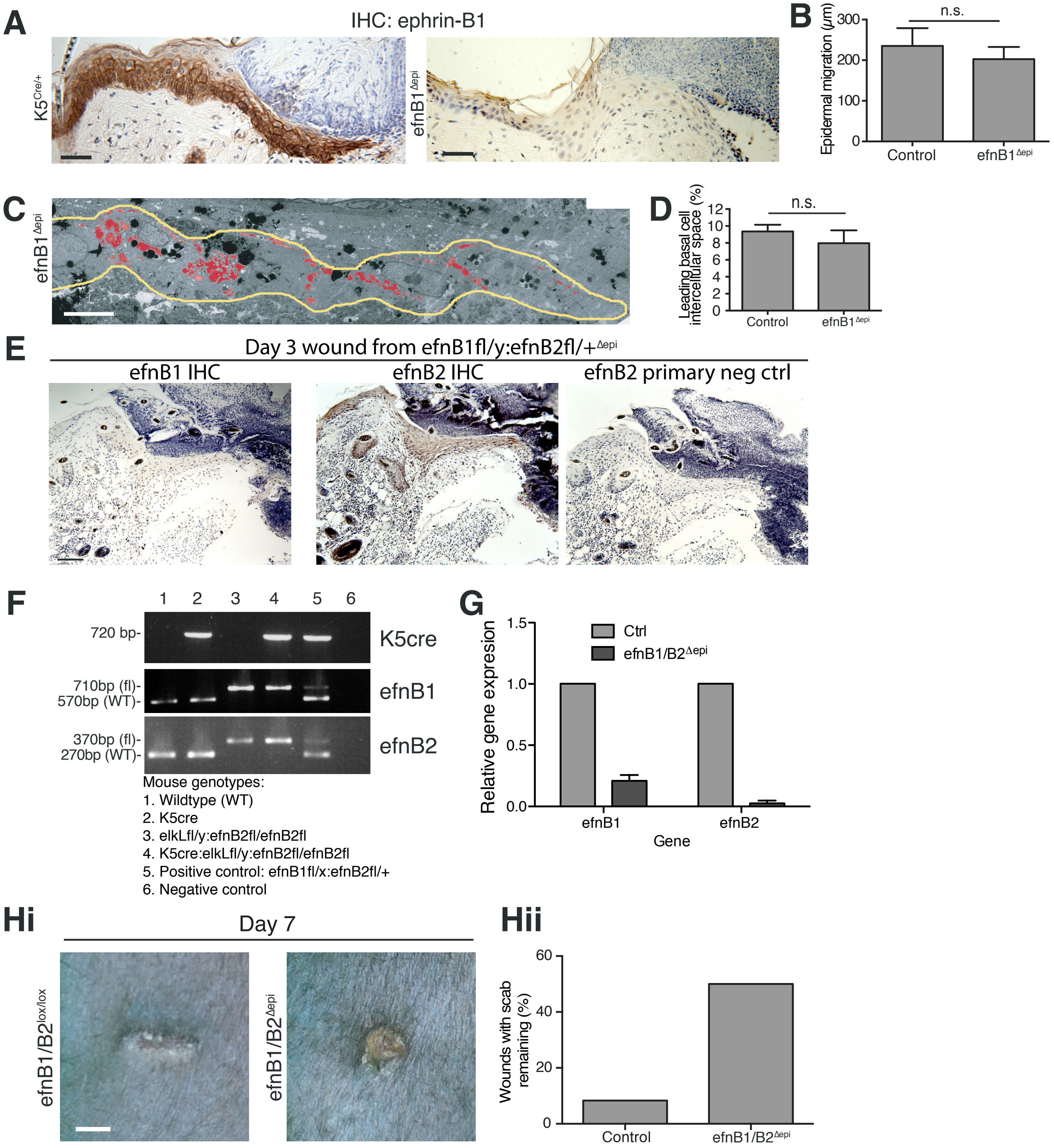

Figure S4, related to Figure 4.

Figure S4, related to Figure 4. Loss of epidermal ephrin-B1 marginally impairs wound healing while epidermal deletion of ephrin-B1 and ephrin-B2 severely inhibits wound healing.

(A) Immunostaining for ephrin-B1 in Control versus keratinocyte-specific ephrin-B1 KO wounds at Day 2 post wounding. (B) Epidermal migration over granulation tissue at Day 2 reveals no significant difference between groups determined by an unpaired Student's t-test. (C) Representative TEM image of the leading edge wound epidermis in efnB1Δepi mice at Day 2 post wounding with basal cells outlined in yellow and intercellular spaces indicated in red. (D) Quantification of intercellular space in Control and efnB1$^{\Delta epi}$ epidermis 2 days post wounding. We see no significant difference as determined by an unpaired Student's t-test, n=2 wounds from 2 mice). (E) Immunostaining reveal that ephrin-B2 (but not B1), is expressed in efnB1$^{\Delta epi}$ migrating epidermal cells. (F) Wild type versus all categories of transgenic mouse DNA confirming genotypes. Key bands indicated for distinguishing floxed versus WT versions of ephrin-B1 and B2. (G) Ephrin-B1 and ephrin-B2 mRNA expression levels compared in Control (efnB1^{lox}efnB2^{lox}) versus KO (efnB1/B2$^{\Delta epi}$) mouse tissues from ear notches (n=4 mice). (H) Control wounds (WT or efnB1^{lox}efnB2^{lox}) versus efnB1/B2$^{\Delta epi}$ were imaged at Day 7 post wounding to determine extent of healing, and the percentage of fully healed wounds determined by scab loss (n=12 wounds from 3 mice). Scale Bars: A = 50μm; C = 10μm; E = 100μm; H = 2mm.

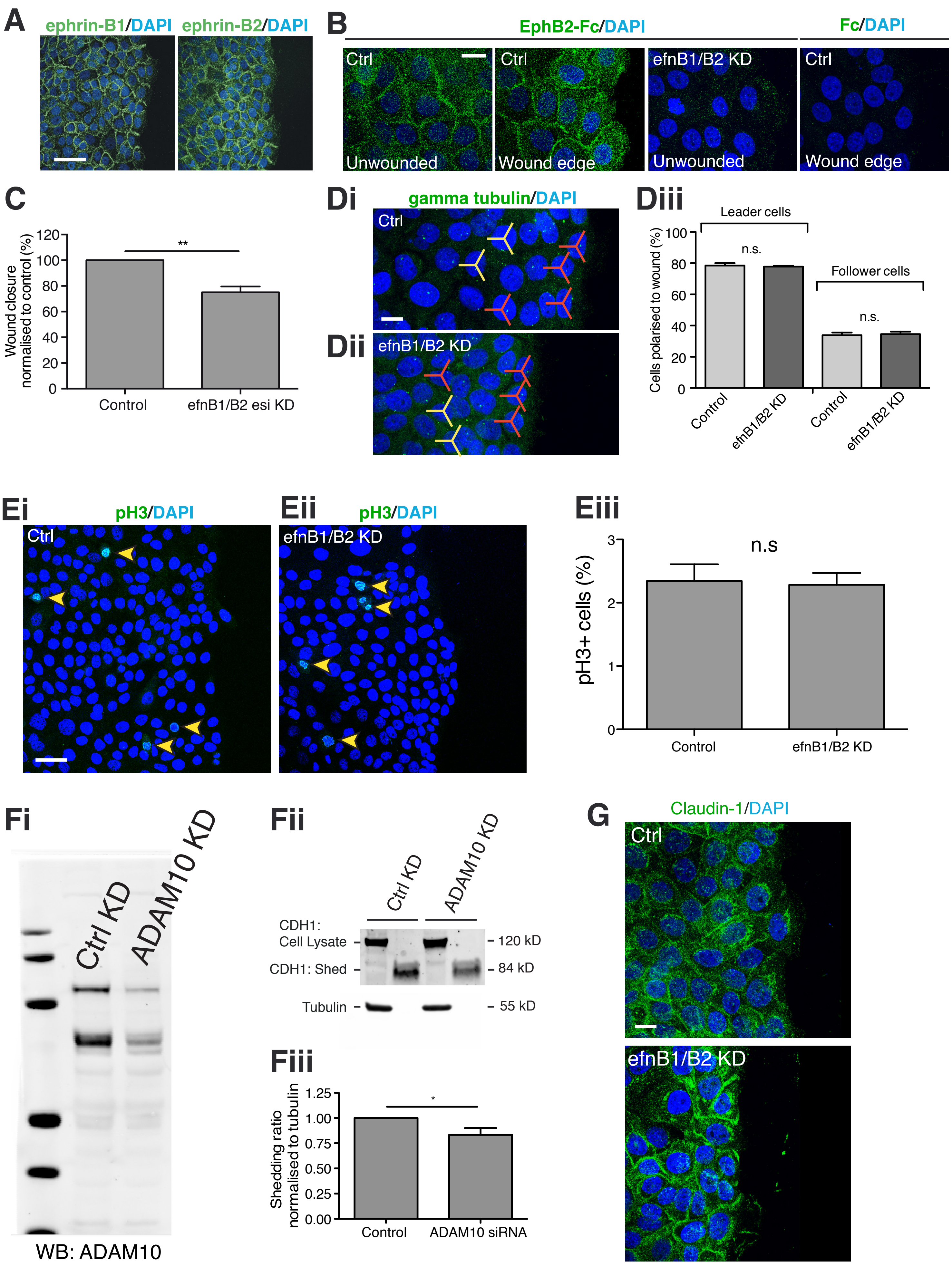

Figure S5, related to Figure 5.

Figure S5, related to Figure 5. Knockdown of ephrin-B1/B2 does not affect HaCaT cell polarity or proliferation and ADAM10 depletion inhibits E-cadherin shedding.
(A) Scratch wounded HaCaT cells reveals membrane staining for both ephrin-B1 and ephrin-B2. (B) Ephrin-B1/B2 localisation similarly can be revealed by staining with Fc-tagged extracellular domain of their shared primary receptor, EphB2-Fc but this staining is lost after siRNA knockdown (KD) in Control unwounded and wounded sheets. (C) Scratch wounded HaCaT cells transfected with a second efnB1/B2 oligo (esiRNA) also show retardation in wound closure (**$P<0.01$, as determined by an unpaired Student's t-test, n=5; 4 replicates per experiment). (D) Extent of wound edge cell polarisation was unaffected in leader or follower cells after ephrinB1/B2 KD (Di and ii), as determined by localisation of the centrosome (gamma tubulin staining) in the front 120° trident (red for polarized cells, yellow for non-polarised). (Diii) Graphic representation of cell polarity data (n.s., $P>0.05$, as determined by an unpaired Student's t-test, n=3; 6 images from 3 coverslips per experiment). (E) Images of pH3 staining (arrowheads) in wounded Control (i) or ephrin-B1/B2 KD (ii) cells. (Eiii) Graph to illustrate lack of effect of ephrinB1/B2 KD on cell proliferation as determined by the percentage of pH3 positive cells (n.s., $P>0.05$, as determined by an unpaired Student's t-test, n=2; 6 images from 3 wells per experiment). (Fi) Western blot for ADAM10 against lysates from Control or ADAM10 siRNA transfected cells. (Fii) Western blot of E-cadherin and tubulin from culture media and cell lysates from Control or ADAM10 KD cells. (Fiii) Graph to illustrate comparison of shed E-cadherin in Control versus ADAM10 KD cells (data within each experiment was normalized to tubulin Control. *$P<0.05$, as determined by an unpaired Student's t-test, n=4). (G) Scratch wounded ephrin-B1/B2 KD cells exhibit elevated membranous Claudin-1 localisation. Scale Bars: A = 50µm; B =20µm; D = 15µm; E=50µm and G = 15µm.

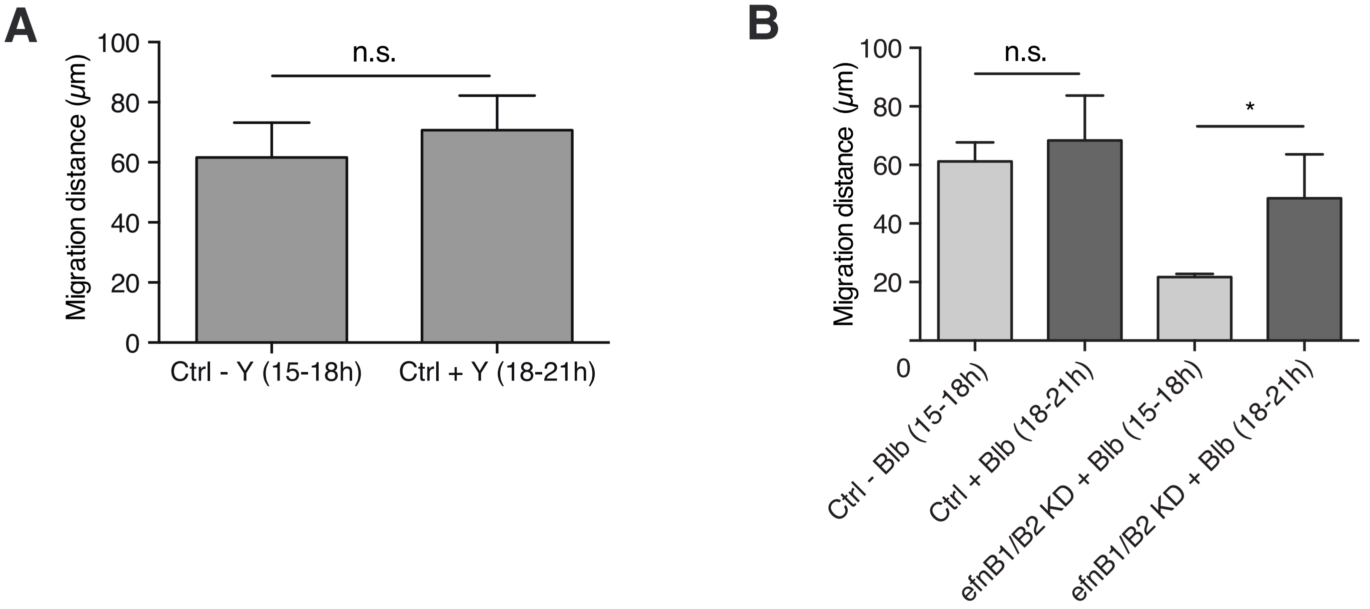

Figure S6, related to Figure 6. Inhibiting actomyosin contractility rescues stalled migration in ephrin-B1/B2 KD HaCaT cells.

(A) Control experiment for Figure 6B. Migration of HaCaT (without ephrin-B1/B2 KD) cells during the 3h before and after Y27632 addition (650nM) at 18h (n=3). This demonstrates that addition of Y27632 (650nM) does not significantly effect migration of Control cells. (B) Migration of Control versus ephrin-B1/B2 KD HaCaT cells from time-lapse images 3h before and after blebbistatin (10µM) treatment (n=3) showing migration surge, just as for Y27632 treatment *P<0.05, as determined by an unpaired Student's t-test.

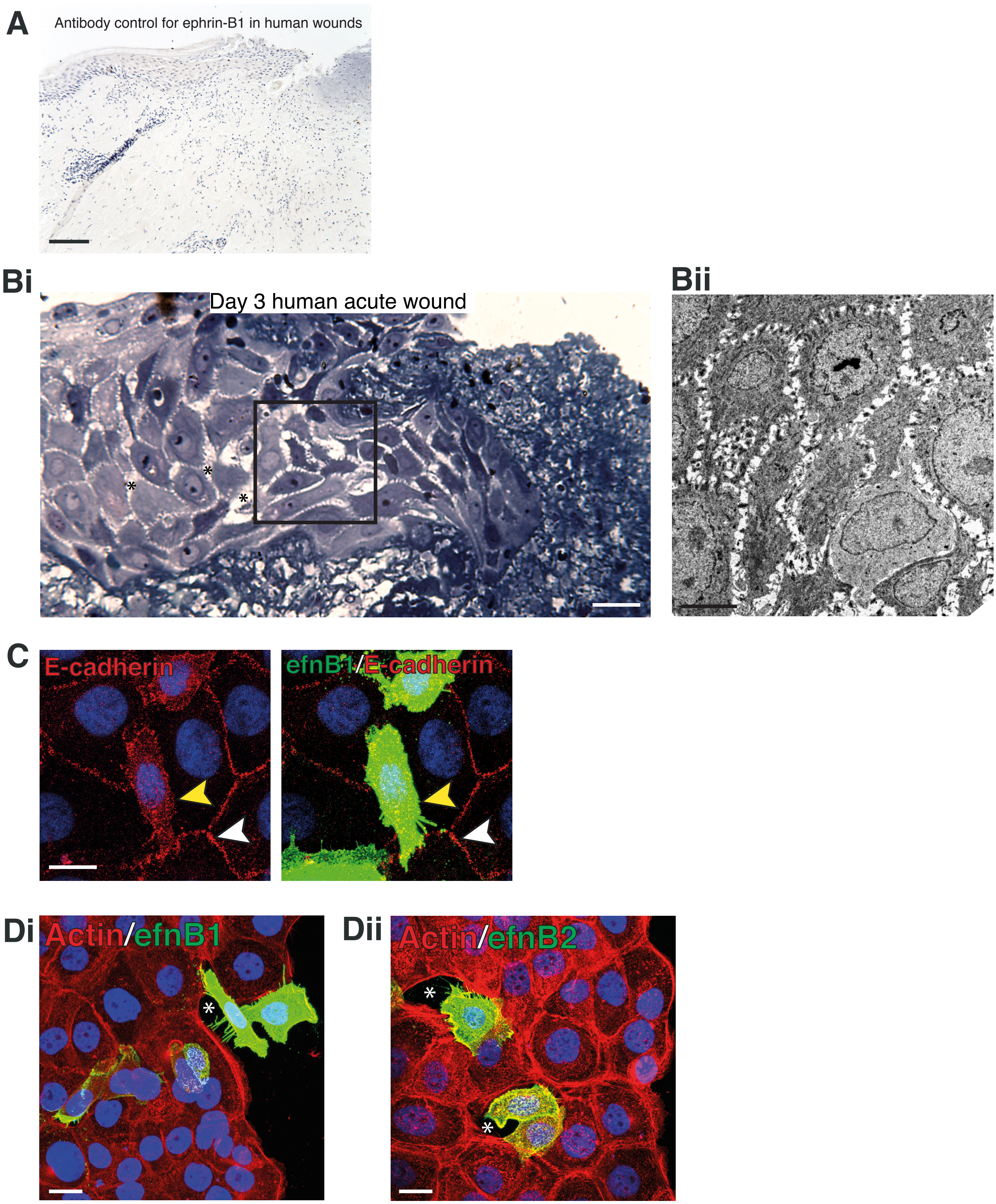

Figure S7, related to Figure 7.

Figure S7, related to Figure 7. Relationship between ephrin-B1/B2 expression and cell:cell adhesion in vitro and human acute wounds.

No primary antibody (negative) control for ephrin-B1 immunostaining in Figure 7C. (Bi) Semi-thin resin section of a Day 3 migrating human acute wound epidermis counterstained with methylene blue showing intercellular spaces (asterisks) in leading edge basal and just suprabasal epidermal cells, just as for mouse wounds. (Bii) TEM illustrating intracellular spaces between basal and suprabasal neighbouring epithelial cells in human wounds; (C) Additional images to those shown in Figure 7E to illustrate how ephrin-B1 overexpressing HaCaT cells lose E-cadherin (red) boundaries between cells (yellow arrow heads), whilst control neighbours retain these adhesions (white arrowheads). (D) Additional images to those shown in Figure 7F to illustrate loss of cell-cell adhesion in cells overexpressing ephrin-B1 (Di), or ephrin-B2 (Dii). Scale Bars: A = 100µm; Bi =100µm; Bii =5µm; C = 15µm and D=20µm.

Supplemental Tables

Table S1, related to Experimental Procedures: Antibodies used in this study.

Primary antibodies					
**Antigen**	**Host**	**Targets**	**Dilution**	**Company**	**Catalogue Code**
ephrin-B1	Goat	Mouse/Human	1:500 (IHC-IF, IHC-DAB, ICC-IF), 1:2000 (WB)	R&D	AF473
ephrin-B2	Rabbit	Mouse/Human	1:50 (IHC-IF, IHC-DAB, IHC), 1:2000 (WB)	Sigma	HPA008999
EphB2	Goat	Mouse/Human	1:250 (IHC-IF)	R&D	AF467
EphB3	Goat	Mouse	1:50 (IHC-IF)	R&D	AF432
Desmoplakin	Mouse	Mouse/Human	1:250 (IHC-IF)	Gift from Prof Garrod	-
E-cadherin	Rat	Mouse/Human	1:500 (IHC-IF, IHC-DAB, ICC-IF), 1:2000 (WB)	Sigma	U3254
Actin (phalloidin-TRITC)	-	F-Actin	1:500	Sigma	P1951
Keratin14	Rabbit	Mouse/Human	1:1000 (IHC-IF)	Covance	PRB-155P
Claudin-1	Rabbit	Mouse/Human	1:5 (IHC-IF, IHC-DAB)	ThermoScientific	RB-9209-R7
g-Tubulin	Mouse	Human	1:500 (IHC-IF)	Serotec	T6557
pH3	Rabbit	Mouse/Human	1:1000 (IHC-DAB, IHC-IF)	Cell Signalling	3377s
Ki67	Rabbit	Mouse/Human	1:2000 (IHC-DAB)	Abcam	Ab15580
ADAM10	Rabbit	Mouse/Human	1:1000 (IHC-DAB), 1:2500 (WB)	Calbiochem	422751

Secondary Antibodies			
**Fluorochrome**	**Target**	**Dilution**	**Company**
Alexa 488	Goat IgG	1:1000 (IF)	Molecular Probes
Alexa 594	Rat IgG	1:1000 (IF)	Molecular Probes
Alexa 488	Mouse IgG	1:1000 (IF)	Molecular Probes
Alexa 488	Rabbit IgG	1:1000 (IF)	Molecular Probes
Alexa 594	Rabbit IgG	1:1000 (IF)	Molecular Probes
Alexa 488	GFP	1:1000 (IF)	Molecular Probes
Dylight 800	Rat IGg	1:5000 (WB)	Molecular Probes
Dylight 680	Rabbit IGg	1:5000 (WB)	Molecular Probes
Dylight 680	Goat IGg	1:5000 (WB)	Molecular Probes

Table S2, related to Experimental Procedures: Mouse qPCR primers used in this study.

Gene	Qiagen Catalogue Number	
**Mouse**		
18s (reference control)	QT02448075	
EFNA1	QT00112364	
EFNA2	QT00097125	
EFNA3	QT01561210	
EFNA4	QT00100681	
EFNA5	QT00116494	
EFNB1	QT00251244	
EFNB2	QT00139202	
EFNB3	QT00147406	
EPHA1	QT00129192	
EPHA2	QT00107534	
EPHA3	QT00129192	
EPHA4	QT00093576	
EPHA5	QT01554756	
EPHA6	QT00171101	
EPHA7	QT00153538	
EPHB1	QT00105987	
EPHB2	QT00089495	
EPHB3	QT00115360	
EPHB4	QT00120295	
EPHB6	QT00156597	
**Human**		
**Gene**	**Forward**	**Reverse**
GAPDH (reference control)	AAGGTCATCCATGACAACTT	ACTGAACCTGACCGTACAAGGCTTCCATTGGATGTT
EFNB1	GAGTTCAGCCCCAACTACAT	ACTGAACCTGACCGTACAAGGCTTCCATTGGATGTT
EFNB2	CTCTGGGGTCTAGAATTTCA	ACTGAACCTGACCGTACAATCTTCATGGCTCTTGTCTG

Table S3, related to Experimental Procedures: RNAi's used in this study.

Gene	Company	Catalogue number
Non target control	Invitrogen (siRNA)	4390843
efnB1	Invitrogen (siRNA)	4392420-s4511
Non target control	Sigma (esiRNA)	EHURLUC
efnB1	Sigma (esiRNA)	EHU035421

Supplemental Movie Legends

Movie S1, related to Figure 5: Time-lapse imaging of Control (top) and ephrin-B1/B2 KD (bottom) HaCaT cells following scratch wounding over 15h (5min/frame). Ephrin-B1/B2 KD cells initially start to migrate before stalling.

Movie S2, related to Figure 5: Time-lapse imaging of Control (top) and ephrin-B1/B2 KD (bottom) HaCaT cells 15h after wounding (2min/frame). Lamellipoda appear normal in the leading edge cells of both Control (top) and ephrin-B1/B2 KD (bottom) monolayers.

Movie S3, related to Figure 6: Time-lapse imaging of ephrin-B1/B2 KD HaCaT cells before and after addition of Y27632 (650nM; indicated by white square at 3h). Y27632 addition temporarily rescues the stalled migration in efnB1/B2 KD cells (5min/frame).

Movie S4, related to Figure 7: Time-lapse imaging of a confluent HaCaT cell monolayer with one efnB1-GFP overexpressing cell (green; 1min/frame) in view.
