# Supplementary figures and images for: Ephrin-Bs Drive Junctional Downregulation and Actin Stress Fiber Disassembly to Enable Wound Re-epithelialization
Source: Cell Rep. 2015 Nov 5;13(7):1380–95. doi: 10.1016/j.celrep.2015.09.085 (PMC4660216; doi:10.1016/j.celrep.2015.09.085)

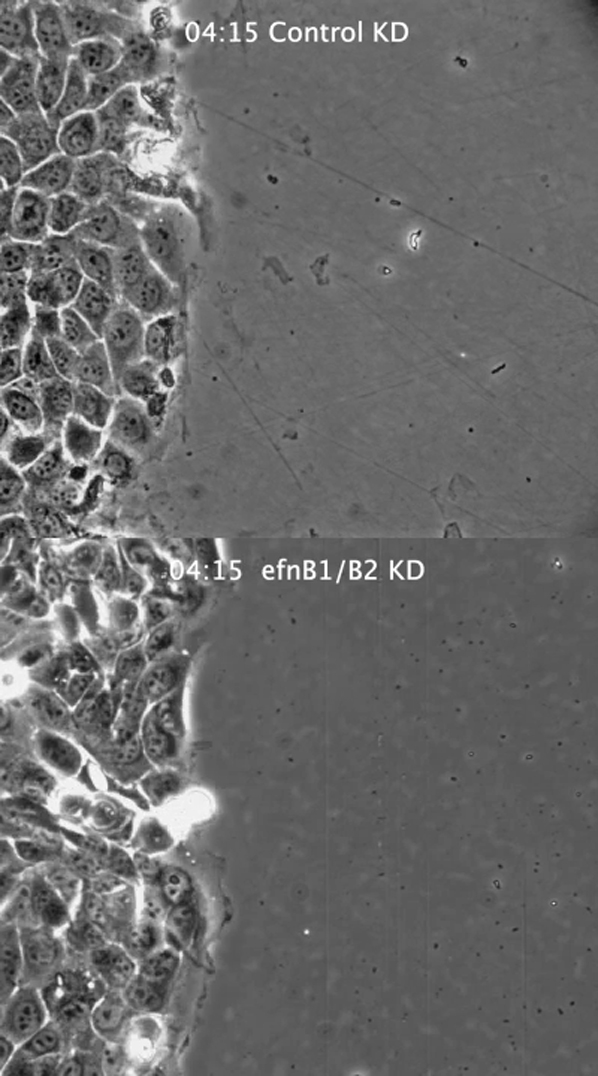

Supplement: Movie S1. Time-Lapse Imaging of Control (top) and Ephrin-B1/B2 KD (bottom) HaCaT Cells following Scratch Wounding over 15 hr (5 min/frame), Related to Figure 5 [file mmc2.jpg]

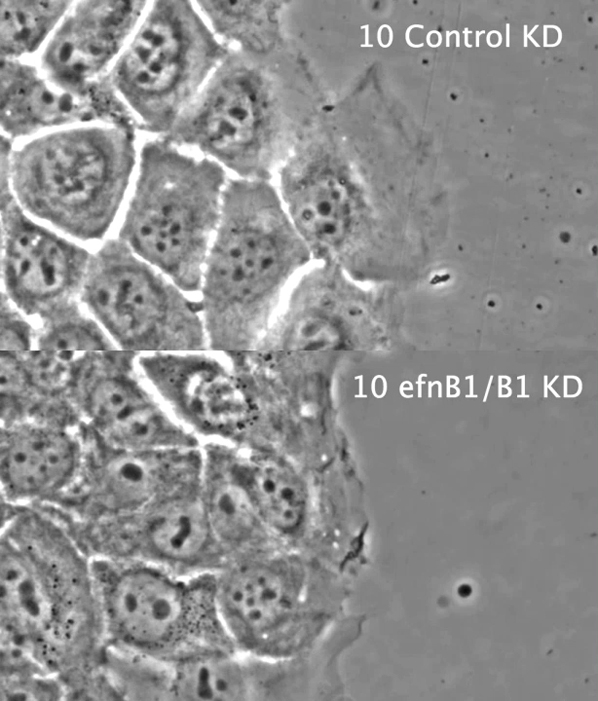

Supplement: Movie S2. Time-Lapse Imaging of Control (top) and Ephrin-B1/B2 KD (bottom) HaCaT Cell Lamellipodia 15 hr after Wounding (2 min/frame), Related to Figure 5 [file mmc3.jpg]

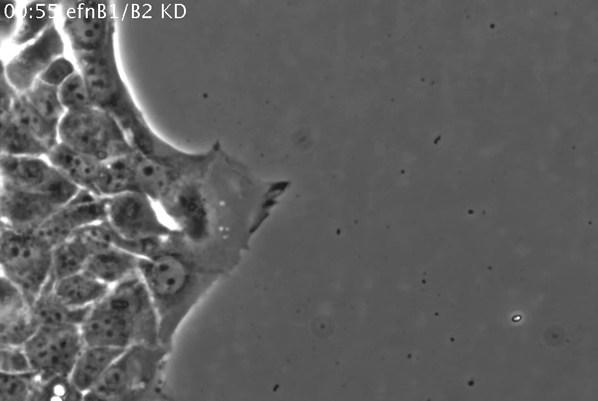

Supplement: Movie S3. Time-Lapse Imaging of Ephrin-B1/B2 KD HaCaT Cells before and after the Addition of Y27632, Related to Figure 6 [file mmc4.jpg]

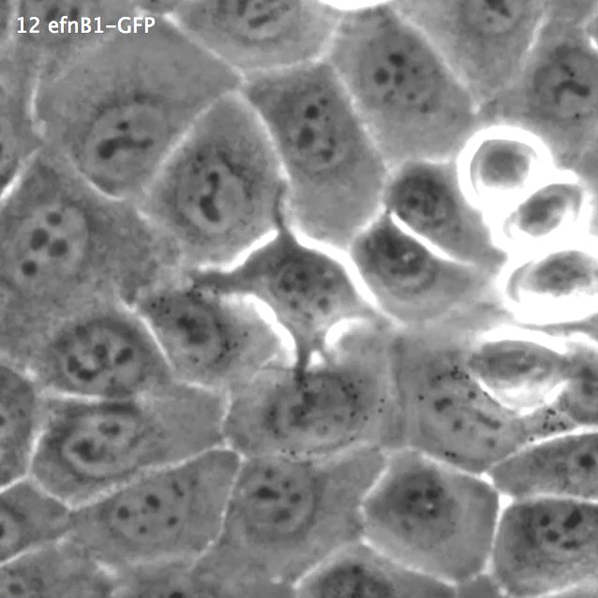

Supplement: Movie S4. Time-Lapse Imaging of a Confluent HaCaT Cell Monolayer with One efnB1-GFP-Overexpressing Cell (green; 1 min/frame) in View, Related to Figure 7 [file mmc5.jpg]
